# Hyperhomocysteinemia, a risk factor for various health conditions: an umbrella review of systematic reviews with meta-analysis

**DOI:** 10.1007/s40520-026-03433-0

**Published:** 2026-07-10

**Authors:** Alberto Castagna, Antonino Maria Cotroneo, Marco Mosele, Pasqualina Sapone, Elisa Martinelli, Masoud Rahmati, Lee Smith, Nicola Veronese

**Affiliations:** 1 Department of Medical and Surgical Sciences, University Magna Græcia, Catanzaro, Italy; 2 Geriatric Unit, Maria Vittoria Hospital, Turin, Italy; 3 Primary Care Department, Unità Locale Socio, Sanitaria 2, Conegliano, Treviso, Italy; 4 Centre d’études Et de Recherche Sur Les Services de Santé Et La Qualité de Vie, Assistance Publique-Hopitaux de Marseille, Aix-Marseille University, 13284 Marseille, France; 5 Centre for Health Performance and Wellbeing, Anglia Ruskin University, Cambridge, UK; 6 Faculty of Medicine, Department of Public Health, Biruni University, Istanbul, Turkey; 7 Faculty of Medicine, Saint Camillus University, Via Sant’Alessandro, 100161 Rome, Italy

**Keywords:** Hyperhomocysteinemia, Chronic kidney disease, Stroke, Dementia, Cardiovascular disease, Meta-analysis, Umbrella review

## Abstract

**Background:**

Hyperhomocysteinemia, an elevation of plasma homocysteine levels, has been implicated in multiple diseases, yet its causal role remains debated. This umbrella review systematically evaluated and graded the evidence from published systematic reviews and meta-analyses on associations between hyperhomocysteinemia and diverse health outcomes.

**Methods:**

We searched Medline, Embase, and Web of Science up to March 5, 2025. Eligible studies were systematic reviews and meta-analyses of observational studies reporting associations between hyperhomocysteinemia and any health outcome. Methodological quality was assessed using AMSTAR-2, and the certainty of evidence was graded using GRADE.

**Results:**

A total of 104 meta-analyses including 2,110 studies and 953,793 participants were analyzed, covering 126 outcomes. Eighty-three percent of outcomes showed significant associations with hyperhomocysteinemia. Nine outcomes were supported by high, 18 by moderate, 73 by low, and 26 by very low certainty of evidence. High-certainty evidence supported associations between hyperhomocysteinemia and increased risks of stroke, intracerebral haemorrhage, fracture (hip and total), and chronic kidney disease. Moderate-certainty evidence linked hyperhomocysteinemia to dementia, Alzheimer’s disease, mortality, and selected cardiovascular and pregnancy outcomes. Most meta-analyses were of low or critically low methodological quality.

**Conclusions:**

Across nearly one million participants, hyperhomocysteinemia was consistently associated with adverse health outcomes, particularly vascular, neurological, skeletal, and renal conditions. While causality cannot be inferred, the evidence supports hyperhomocysteinemia as a potentially modifiable risk factor meriting targeted preventive and interventional research.

**Protocol registration:**

https://osf.io/dezms

**Supplementary Information:**

The online version contains supplementary material available at https://doi.org/10.1007/s40520-026-03433-0.

## Introduction

Homocysteine (Hcy) is a sulfur-containing amino acid formed during the metabolism of methionine, an essential amino acid derived from dietary protein. [1] Its plasma concentration reflects the balance between its production and clearance through two main pathways: remethylation to methionine—dependent on folate and vitamin B₁₂ and catalyzed by methylenetetrahydrofolate reductase (MTHFR)—and transsulfuration to cysteine via cystathionine β-synthase (CBS). [1] Disruption of these enzymatic reactions can lead to an abnormal increase in circulating Hcy, a condition known as hyperhomocysteinemia, typically defined as plasma Hcy levels exceeding 15 μmol/L. [2].

Since McCully’s seminal observation in 1969 linking homocystinuria to vascular pathology, elevated Hcy has been increasingly recognized as a potential risk factor for a wide range of clinical conditions, including cardiovascular and cerebrovascular diseases, cognitive decline, as well as metabolic and renal disorders. [3] Epidemiological studies have demonstrated that even moderate elevations in Hcy are associated with an increased risk of stroke [4], coronary artery disease [5], and dementia. [6].

Nutritional factors, particularly folate and vitamin B₁₂, play central roles in regulating Hcy metabolism. [1] Low levels of these vitamins are inversely correlated with plasma Hcy concentration, and supplementation with folic acid and related B vitamins has been shown to effectively reduce Hcy levels. [7] Randomized controlled trials (RCTs) and meta-analyses have suggested that Hcy-lowering interventions may have protective effects, especially against stroke, though evidence for other outcomes such as cancer or dementia remains inconsistent. [7].

Despite decades of research, the clinical significance of Hcy continues to be debated. The magnitude, consistency, and causality of Hcy–disease relationships have been variably reported across studies, and no consensus has been reached regarding whether Hcy is an independent risk factor or a biomarker of other underlying metabolic disturbances. [8] Moreover, although asymptomatic Hcy is common, it is not currently regarded as an indication for pharmacological intervention in clinical practice. [9].

To clarify these uncertainties, comprehensive approaches that integrate evidence from multiple study designs are essential.

The present umbrella review aims to systematically evaluate and appraise the available evidence on associations between hyperhomocysteinemia and a wide range of health outcomes. By summarizing data from previously published systematic reviews and meta-analyses, and applying validated frameworks for credibility assessment, this work seeks to determine the certainty and consistency of evidence linking elevated Hcy levels to major diseases, thereby providing insight into potential preventive and therapeutic implications for clinical and public health practice.

## Methods

### Protocol and registration

This systematic review was conducted following the recommendations of the Cochrane handbook for systematic literature reviews to carry out the screening and selection of studies and reported according to the updated 2020 Preferred Reporting Items for Systematic Review and Meta-Analysis (PRISMA) guidelines [10, 11]. The protocol is freely available in Open Science Framework (https://osf.io/dezms).

## PICO question and eligibility criteria

Following the PICOS (participants, intervention, control, outcomes, study design) question, we included:

- Participants: any.
- Exposure: hyperhomocysteinemia, independently from the definition, using highest or as linear increase.
- Controls: lowest category.
- Outcomes: all health outcomes.
- Study design: systematic reviews and meta-analyses of observational studies.

We excluded the following studies: (i) meta-analyses of intervention studies, e.g., reporting intervention to lowering homocysteinemia levels; (ii) meta-analyses exploring genetic factors commonly associated with high homocysteinemia levels.

## Information sources and search strategies

For this umbrella review, several relevant bibliographic databases were comprehensively searched, including Medline (via Ovid), Embase, and Web of Science from database inception up to the 5th March 2025. Full details of the search strategy are provided in Supplementary Table 1.

## Study selection

Using Covidence [12], the selection of potentially eligible works was independently carried, in doubles, by several review authors (MR, AC, EM, PS), with consensus meetings to discuss the studies for which divergent selection decisions were made by the two review authors. A third senior member of the review team (NV) was involved, if necessary. The studies selection process involved, first, a selection based on title and/or abstracts, then a selection of studies retrieved from this first step based on the full-text manuscripts.

## Data collection and data items

From the eligible full-text articles we extracted: first author name and affiliation, year of publication, journal name, title of the manuscript; data on the characteristics of the population considered, sample size, study design (case–control or cohort), definition of hyperhomocysteinemia (highest vs. lowest category or as a linear increase), outcome of interest, number of studies included, number of events (in case–control studies was the number of people having the condition of interest, in cohort studies the number of people maturating the condition during the follow-up period). The data regarding estimates were extracted at meta-analysis level and categorized in risk ratio (RR), odds ratio (OR), hazard ratio (HR), mean difference (MD) or standardized MD (SMD). These data were collected using a standardized Excel data extraction form. The data extraction was led by one review author (MR, AC, EM, PS) and systematically double checked by a second independent review author. Errors found in extraction by the second author review were corrected during a consensus meeting by both authors, and consulted with a senior author when a decision could not be reached (NV).

## Assessment of risk of bias

One author (MM) rated the methodological quality of the included systematic reviews using “A MeaSurement Tool to Assess systematic Reviews 2 (AMSTAR 2)” [13], which ranks the quality of a meta-analysis in one of 4 categories ranging from “critically low” to “high” according to 16 predefined items. [13] Another author (NV) double checked this evaluation.

## Data synthesis and grading of the evidence

The evidence from meta-analyses was evaluated using the GRADE (Grading of Recommendations, Assessment, Development and Evaluation) assessment. The GRADE framework takes into account several important domains for the judgment of the certainty of the evidence, including study design, risk of bias, inconsistency, indirectness, imprecision and other aspects, such as publication bias or strong association between the exposure (i.e., hyperhomocysteinemia) and the outcomes of interest. [14] Supplementary Table 2 reports the criteria used, for each domain, for assessing the GRADE. The only deviation from the original GRADE was the fact that we did not undergrade the level for observational studies compared to randomized controlled trials, since our umbrella review included only meta-analyses of observational studies. The certainty of the evidence was then categorized as very low (the true effect is probably markedly different from the estimated effect), low (the true effect might be markedly different from the estimated effect), moderate (the true effect is probably close to the estimated effect) or high (there is a lot of confidence that the true effect is similar to the estimated effect). [14].

## Ethical issues

This study did not involve patients or any human or animal material, and therefore does not imply any ethical issue.

## Results

### Literature search

**Fig. 1:**
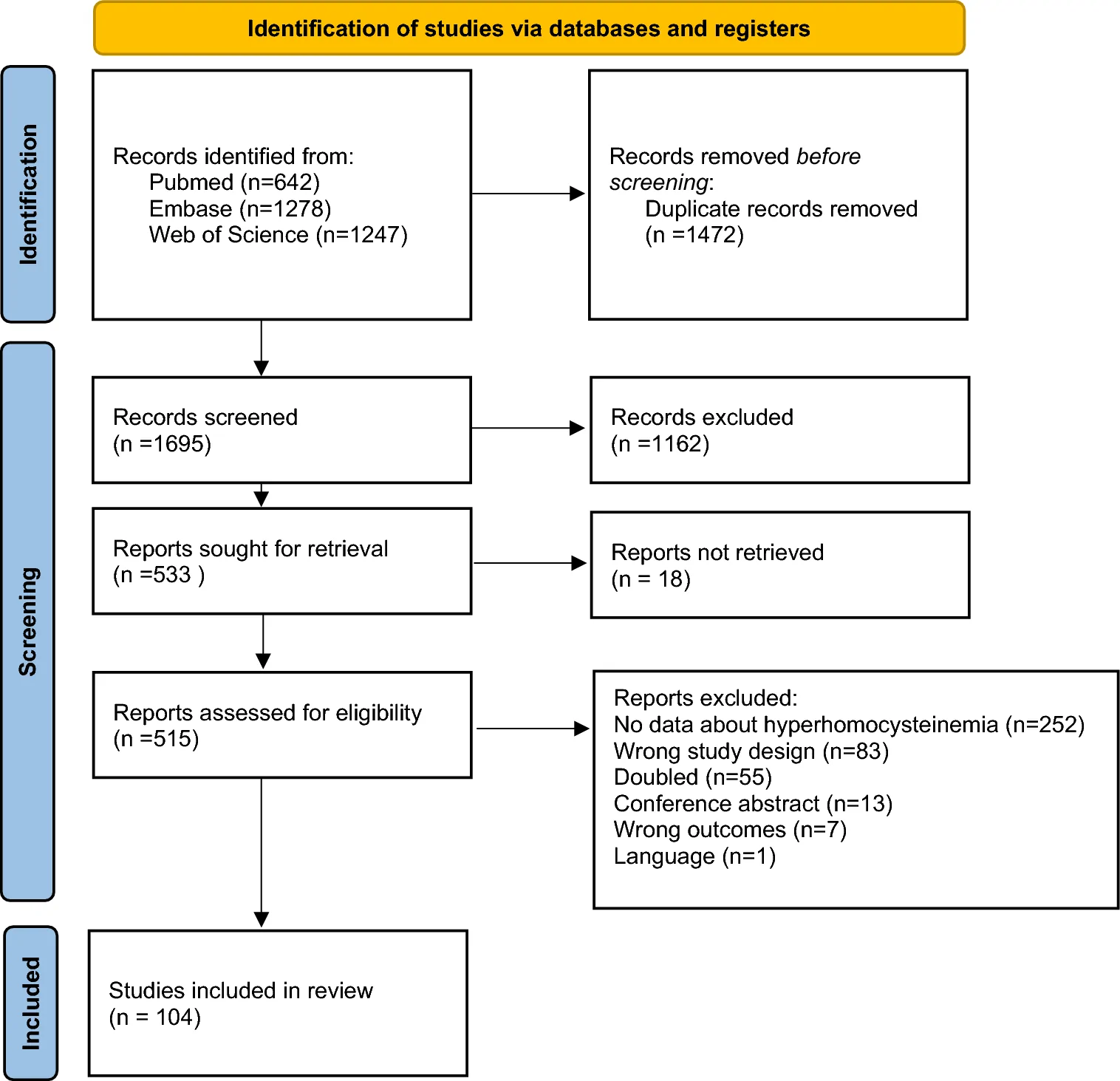
PRISMA flow

As shown in Fig. 1, among 3,167 papers initially screened, we evaluated 515 full-texts. After excluding 411 full-texts, mainly owing to them not reporting data on hyperhomocysteinemia (n = 252) or were not systematic reviews with meta-analyses (n = 82), we included 104 systematic reviews and meta-analyses. The list of works excluded at full-text level is reported, with the main reason, in Supplementary Table 3, whilst the list of the 104 included works are reported in Supplementary Table 4.

## Descriptive findings of the umbrella review

The 104 meta-analyses gave data for 126 independent outcomes divided according to their nature in Tables 1, 2, 3, 4, 5, 6, 7, 8, and 9. Overall, 2,110 studies, mainly case–control, for a total of 953,793 people were included. The main number of studies included for each outcome was 17 and the mean number of participants was 8,671. Among the 126 outcomes analyzed, 105 (= 83.3%) reported a statistically significant association between hyperhomocysteinemia and the outcome of interest. Overall, nine outcomes were supported by a high level of evidence, 18 by a moderate, 73 by a low level of evidence, and 26 by a very low level of evidence.

**Table 1:** Evidence about hyperhomocysteinemia and cancer-related outcomes

Author	Year	Type Of Population	Type Of Study	Definition Of Hyperomocisteinemia	Outcome	N° Studies For The Outcome	Number Of Events	Total Sample Size (Outcome)	Type Of Effect Size	Effect Size	95% Ci Low—Outcome	95%Ci High—Outcome	% Studies At High Risk Of Bias	Heterogeneity	Indirectness	Imprecision	Publication Bias	Large Effect	GRADE
Xu	2018	General population	Case–control	Highest vs lowest category	Digestive tract cancer	13	NA	NA	OR	1.27	1.15	1.39	< 33%	0	No	Yes	No	No	Moderate
Yang	2018	General population	Case–control	Highest vs lowest category	Lung cancer	8	NA	NA	SMD	0.41	0.24	0.58	< 33%	61.9	No	No	NA	No	Moderate
Collin	2010	Men 50–69 years	Case–control	Linear increase	prostate cancer	20	1461	2968	OR	1.17	0.95	1.43	NA	11.1	No	No	NA	No	Low
Zhang	2015	General population	Case–control	Highest vs lowest category	Cancer	84	15,046	20,712	OR	5.06	4.59	5.5	< 33%	90	No	No	Yes	Very large effect	Low
Niu	2023	General population	Case–control	Linear increase	SSNHL	9	565	1718	SMD	0.41	0.11	0.72	NA	74	No	No	NA	No	Very low

Abbreviations

**Table 2:** Evidence about hyperhomocysteinemia and cardiovascular-related outcomes

Author	Year	Type Of Population	Type Of Study	Definition Of Hyperomocisteinemia	Outcome	N° Studies For The Outcome	Number Of Events	Total Sample Size (Outcome)	Type Of Effect Size	Effect Size	95% Ci Low—Outcome	95%Ci High—Outcome	% Studies At High Risk Of Bias	Heterogeneity	Indirectness	Imprecision	Publication Bias	Large Effect	GRADE
Cao	2014	General population	Case–control	Linear increase	aortic aneurysm	7		6445	OR	3.29	1.66	6.51	NA	3.29	No	No	NA	Very large effect	Moderate
Huang	2017	General population	Cohort	Highest vs lowest category	Retinal artery occlusion	6	307	542	OR	6.64	3.42	12.89	< 33%	57	No	Serious	No	Very large effect	Moderate
Verkleij-Hagoort	2007	General population	Case–control	Linear increase	congenital heart defects	5	1341	16,925	OR	4.4	2.6	7.3	NA	15.2	No	No	No	Very large effect	Moderate
Zhang	2019	General population	Case–control	Highest vs lowest category	non fatal MI	19		4340	RR	1.36	0.89	2.09	NA	18.2	No	No	No	No	Moderate
Zhang	2019	General population	Case–control	Highest vs lowest category	Restenosis	19		4340	RR	1.1	0.9	1.33	NA	49.1	No	No	No	No	Moderate
Zhang	2019	General population	Case–control	Highest vs lowest category	cardiac death	19		4340	RR	2.76	1.44	5.32	NA	24.3	No	No	No	Large effect	Moderate
Bautista	2002	General population	Case–control	Linear increase	first cardiac event	13		9834	OR	1.49	1.31	1.7	NA	p < 0.05	No	No	NA	No	Low
Boushey	1995	General population	Case–control	Linear increase	Coronary artery disease	30		NA	OR	1.7	1.5	1.9	NA	p < 0.05	No	No	NA	No	Low
Boushey	1996	General population	Case–control	Linear increase	peripheral arterial disease	5		NA	OR	6.8	2.9	15.8	NA	p < 0.05	No	No	NA	Very large effect	Low
Clarke. Homocystine Studies Collaboration	2002	Healthy Population	Cohort	Linear increase	stroke	30	1113	16,786	OR	0.81	0.69	0.95	NA	p < 0.05	No	No	NA	No	Low
Clarke. Homocystine Studies Collaboration	2002	Healthy Population	Cohort	Linear increase	Ischemic heart disease	30	5073	16,786	OR	0.89	0.83	0.96	NA	p < 0.05	No	No	NA	No	Low
denHeijer	1998	General population	Case–control	Linear increase	venous thrombosis	10	1144	2620	OR	2.5	1.8	3.5	NA	NA	No	No	NA	Large effect	Low
Dong	2021	General population	Case–control	Linear increase	atrial Fibrillation	5	5790	15,556	SMD	0.81	0.58	1.03	33–66%	73	No	No	No	Very large effect	Low
Habib	2023	General population	Case–control	Linear increase	coronary artery disease	38	1421	3617	SMD	0.877	0.792	0.962	NA	97.98	No	No	NA	Very large effect	Low
Heinz	2009	patients with end-stage renal disease	Case–control	Linear increase	CVD events	9	631	1506	HR	1.09	1.03	1.14	NA	48.5	No	No	NA	No	Low
Jin	2021	General population	Case–control	Linear increase	Heart failure	12	1530	5506	SMD	1.15	0.71	1.58	< 33%	96.6	No	No	Yes	Very large effect	Low
Khandanpour	2009	General population	Case–control	Linear increase	peripheral arterial disease	14	2365	6599	MD	4.31	1.71	6.91	NA	98	No	No	NA	No	Low
La Regina	2010	Behcet disease patients	Case–control	Linear increase	venous and arterial thrombosis	16	153	327	OR	3.14	1.26	7.8	> 66%	63	No	Serious	No	Very large effect	Low
Luo	2014	General population	Case–control	Linear increase	Cervical artery dissection	5	271	608	SMD	0.96	0.42	1.49	< 33%	88.8	No	Serious	Yes	Very large effect	Low
Merashli	2022	Smokers	Case–control	Linear increase	Buerger’s disease	3	61	118	SMD	0.401	0.01	0.792	NA	11.40	No	Serious	NA	No	Low
Merashli	2022	General population	Case–control	Linear increase	Buerger’s disease	7	193	621	SMD	0.863	0.675	1.05	NA	16.80	No	Serious	NA	Very large effect	Low
Peng	2015	General population	Cohort	Linear increase	CHD	12	2396	23,623	RR	1.66	1.12	2.47	< 33%	85.9	No	No	No	No	Low
Piao	2018	General population	Case–control	Linear increase	Cerebral Small Vessel Disease	18	1987	5088	SMD	0.5	0.36	0.64	< 33%	80.7	No	No	No	Large effect	Low
Ray	1998	General population	Case–control	Linear increase	TEV	9	856	2409	OR	2.95	2.08	4.17	NA	NA	No	No	NA	Large effect	Low
Unadkat	2024	General population	Case–control	Linear increase	CAD	59	9381	21,569	SMD	0.73	0.55	0.91	< 33%	94	No	No	Yes	Very large effect	Low
Wald	2004	General population	Cohort	Linear increase	Ischaemic heart disease	20	3144		OR	1.42	1.11	1.84	NA	NA	No	No	NA	No	Low
Wu	2018	General population	Case–control	Highest vs lowest category	calcific aortic valve disease	10	NA	6349	SMD	0.57	0.36	0.79	< 33%	91	No	No	No	Large effect	Low
Zhong	2017	General population	Case–control	Highest vs lowest category	essential hypertention	11	4830	16,571	OR	1.36	1.02	1.8	< 33%	82.8	No	No	No	No	Low
Cahill	2003	General population	Case–control	linear increase	retinal vascular occlusive disease	19	614	1376	OR	0.86	0.73	0.99	NA	NA	No	No	NA	No	Very low
Danesh	1998	General population	Case–control	Linear increase	heart disease	22	840	2127	OR	1.9	1.6	2.3	NA	NA	No	No	NA	No	Very low
Rong	2020	General population	Case–control	Linear increase	FA	11	3974	9334	OR	2.21	1.16	4.21	NA	94.5	No	No	NA	Large effect	Very low

Abbreviations

**Table 3:** Evidence about hyperhomocysteinemia and metabolic-related outcomes, including osteoporosis

Author	Year	Type Of Population	Type Of Study	Definition Of Hyperomocisteinemia	Outcome	N° Studies For The Outcome	Number Of Events	Total Sample Size (Outcome)	Type Of Effect Size	Effect Size	95% Ci Low—Outcome	95%Ci High—Outcome	% Studies At High Risk Of Bias	Heterogeneity	Indirectness	Imprecision	Publication Bias	Large Effect	GRADE
He	2021	Older adults	Case–control	Linear increase	fracture	28	NA	47,704	HR	1.25	1.12	1.4	< 33%	45	No	No	No	No	High
Yang	2012	General population	Cohort	Highest vs lowest category	Fracture	9	1457	14,863	RR	1.59	1.3	1.96	< 33%	48.5	No	No	No	No	High
Xu	2014	Diabetes	Cohort	Highest vs lowest category	Diabetic retinopathy	9	2070	5547	RR	1.93	1.46	2.53	< 33%	26.9	No	No	No	No	High
Yang	2012	General population	Cohort	Highest vs lowest category	Hip fracture	4	NA	14,683	RR	1.67	1.17	2.38	< 33%	58.2	No	No	No	No	Moderate
Huang	2013	General population	Case–control	Linear increase	DM2	14	4011	4303	SMD	0.94	0.4	1.48	NA	98	No	No	No	Very large effect	Low
Zheng	2015	Diabetes	Case–control	Linear increase	progression of DPN	6	603	1290	SMD	1.23	1.09	1.36	NA	98.8	No	No	No	Very large effect	Low
Mao	2014	Diabetes	Case–control	Linear increase	type 2 diabetic nephropathy macro-albuminuria	3	47	143	SMD	0.99	0.62	1.36	> 66%	0	No	Serious	No	Very large effect	Low
Mao	2014	Diabetes	Case–control	Linear increase	type 2 diabetic nephropathy mIcro-albuminuria	6	305	914	SMD	1.29	0.59	2	> 66%	97.4	No	Serious	No	Very large effect	Low
Sansone	2019	General population	Case–control	Linear increase	DE	9	489	1320	SMD	1	0.65	1.35	NA	85.3	No	No	NA	Very large effect	Low
Ulloque-Badaracco	2024	General population	Case–control	Linear increase	polycystic ovary syndrome	75	4453	52,230	SMD	0.82	0.62	1.02	< 33%	94	No	No	Yes	Very large effect	Low
Wang	2021	General population	Case–control	Linear increase	Obesity	14	710	1317	SMD	0.76	0.25	1.27	< 33%	94	No	No	No	Very large effect	Low
Zhou	2014	General population	Case–control	Highest vs lowest category	severity of hypothyroidism	17		NA	SMD	0.19	-0.07	0.45	> 66%	62.9	No	No	No	No	Low
Cheng	2024	General population	Case–control	Linear increase	erectile dysfunction	12		4106	OR	0.97	0.52	1.43	< 33%	97	No	No	No	No	Very low
Cheng	2022	Chinese general population	Case–control	Linear increase	type 2 DM	1	370	772	OR	1.032	1.003	1.006	NA	88	No	Serious	NA	No	Very low
Costa	2021	NAFLD	Case–control	Linear increase	NAFLDO	8	361	871	MD	1.95	0.74	3.15	< 33%	77	No	Serious	NA	No	Very low
Feng	2015	General population	Case–control	Linear increase	DM1	15	592	913	SMD	-0.08	-0.44	0.28	> 66%	84.9	No	Serious	Yes	No	Very low
Mao	2014	Diabetes	Case–control	Linear increase	type 2 diabetic nephropathy macro-albuminuria	4	97	706	SMD	1.66	0.46	2.87	> 66%	93.1	No	Serious	No	Very large effect	Very low
Pinna	2018	General population	Case–control	Linear increase	Wet AMD	12	453	967	MD	1.1	0.96	1.25	33–66%	91.8	No	Serious	Yes	No	Very low
Ulloque-Badaracco	2023	Metabolic syndrome	Case–control	Linear increase	metabolic syndrome	66		87,988	OR	0.87	0.81	0.93	< 33%	90	No	No	Yes	No	Very low
Yuan	2022	General population	Case–control	Highest vs lowest category	NAFLD	20	NA	NA	OR	1.96	1.57	2.45	< 33%	89	No	No	No	No	Very low
Zhang	2020	patients with subclinical hypothyroidism	Case–control	Highest vs lowest category	severity of hypothyroidism	17	NA	NA	SMD	0.19	-0.07	0.45	> 66%	62.9	No	No	No	No	Very low

Abbreviations:

**Table 4:** Evidence about hyperhomocysteinemia and eye-related outcomes

Author	Year	Type Of Population	Type Of Study	Definition Of Hyperomocisteinemia	Outcome	N° Studies For The Outcome	Number Of Events	Total Sample Size (Outcome)	Type Of Effect Size	Effect Size	95% Ci Low—Outcome	95%Ci High—Outcome	% Studies At High Risk Of Bias	Heterogeneity	Indirectness	Imprecision	Publication Bias	Large Effect	GRADE
LI	2016	General population	Case–control	Linear increase	Normal-Tension Glaucoma	4	149	297	MD	0.01	-0.13	2.45	NA	72	No	Serious	NA	No	Low
Huang	2015	General population	Case–control	Linear increase	risk for Age-related macular degeneration	11	1072	1202	MD	2.67	1.6	3.74	NA	92	No	No	No	No	Very low
Xu	2012	General population	Case–control	Highest vs lowest category	Pseudoexfoliation glaucoma	14	941	485	MD	3.38	2.35	4.42	NA	73.4	No	Serious	No	No	Very low
Xu	2012	General population	Case–control	Highest vs lowest category	Primary Open-Angle Glaucoma	7	430	847	MD	2.28	0.41	4.14	NA	96	No	Serious	NA	No	Very low

Abbreviations

**Table 5:** Evidence about hyperhomocysteinemia and mortality

Author	Year	Type Of Population	Type Of Study	Definition Of Hyperomocisteinemia	Outcome	N° Studies For The Outcome	Number Of Events	Total Sample Size (Outcome)	Type Of Effect Size	Effect Size	95% Ci Low—Outcome	95%Ci High—Outcome	% Studies At High Risk Of Bias	Heterogeneity	Indirectness	Imprecision	Publication Bias	Large Effect	GRADE
Fan	2017	General population	Cohort	Linear increase	all cause mortality	11	4110	27,737	RR	1.88	1.51	2.14	< 33%	64.8	No	No	No	No	Moderate
Ulloque-Badaracco	2024	Patients with COVID-19	Case–control	Linear increase	Mortality	4		534	SMD	1.69	-2.39	5.76	NA	99.4	No	Serious	No	Very large effect	Low
Zhang	2019	PCI	Case–control	Highest vs lowest category	all cause mortality	19		4340	RR	3.19	1.9	5.34	NA	55.6	No	No	No	Very large effect	Low
Lu	2025	Sepsis	Cohort	Linear increase	Death	9	212	771	SMD	-0.23	-0.84	0.37	< 33%	86	No	Serious	No	No	Very low
Yuan	2022	Intensive care unit	Case–control	Highest vs lowest category	Mortality	13	695	985	MD	-3.42	-5.89	-0.94	< 33%	86	No	Serious	No	No	Very low

Abbreviations:

**Table 6:** Evidence about hyperhomocysteinemia and neurological-related outcomes

Author	Year	Type Of Population	Type Of Study	Definition Of Hyperomocisteinemia	Outcome	N° Studies For The Outcome	Number Of Events	Total Sample Size (Outcome)	Type Of Effect Size	Effect Size	95% Ci Low—Outcome	95%Ci High—Outcome	% Studies At High Risk Of Bias	Heterogeneity	Indirectness	Imprecision	Publication Bias	Large Effect	GRADE
Jaiswal	2024	Stroke	Case–control	Linear increase	post-stroke depression	12	771	2335	SMD	0.14	0.05	0.22	< 33%	0	No	No	No	No	High
Li	2022	General population	Cohort	Linear increase	hemorrhagic stroke	4	625	4978	OR	1.99	1.03	3.84	< 33%	32.6	No	No	NA	No	High
Li	2022	General population	Cohort	Linear increase	ischemic stroke	13	1745	27,125	OR	1.71	1.39	2.11	< 33%	45.2	No	No	NA	No	High
Zhou	2018	General population	Case–control	Highest vs lowest category	Intracerebral hemorrhage	7	667	2488	SMD	0.59	0.51	0.68	< 33%	0	No	No	No	Large effect	High
Li	2022	General population	Cohort	Linear increase	first time stroke	15	2084	32,269	OR	1.95	1.59	2.37	< 33%	55.7	No	No	NA	No	Moderate
Li	2022	General population	Cohort	Linear increase	stroke	24	4983	51,426	OR	1.95	1.59	2.37	< 33%	56.7	No	No	NA	No	Moderate
Liampas	2020	Migraine	Case–control	Linear increase	Migrain with aura	10	579	138	MD	0.3	0.14	0.46	NA	39	No	Serious	NA	No	Moderate
Wald. D. S	2011	General population	Cohort	Linear increase	dementia	8	908	8669	OR	1.5	1.13	2	NA	0	No	No	No	No	Moderate
Wang	2021	General population	Case–control	Highest vs lowest category	Dementia	56	NA	NA	SMD	0.81	0.69	0.94	< 33%	85.2	No	No	Yes	Very large effect	Moderate
Zhou	2019	General population	Cohort	Highest vs lowest category	Alzheimer-type dementia	28	2557	28,257	RR	1.15	1.04	1.26	< 33%	56.6	No	No	No	No	Moderate
Bautista	2002	General population	Case–control	Linear increase	First cerebrovascolar event	1	58	878	OR	1.37	0.99	1.88	NA	p < 0.05	No	Serious	NA	No	Low
Ford	2002	General population	Case–control	Linear increase	cerebrovascular disease	24	1817	6604	OR	1.23	1.07	1.41	NA	NA	No	No	NA	No	Low
Fu	2015	General population	Case–control	Linear increase	cerebral infarction	13	1206	2408	SMD	1.324	0.813	1.835	< 33%	96.78	No	No	No	Very large effect	Low
Guo	2020	General population	Cohort	Linear increase	Autism	31	1641	3304	SMD	0.56	0.36	0.76	< 33%	87	No	No	No	Large effect	Low
Ho	2011	General population	Cross-sectional	Linear increase	Developing dementia/ cognitive decline	17	1455	6122	OR	0.56	0.94	1.91	NA	36.7	No	No	Yes	Large effect	Low
Hu	2022	General population	Case–control	Linear increase	ALS	9	812	2632	MD	1.56	-0.07	3.19	NA	94	No	No	NA	No	Low
Zhang	2020	Chinese general population	Case–control	Linear increase	ischemic stroke	13	871	3114	SMD	1.15	0.85	1.45	< 33%	89.9	No	No	No	Very large effect	Low
LI	2019	General population	Case–control	Linear increase	Multiple sclerosis	17	1419	2624	SMD	0.64	0.33	0.95	NA	92	No	No	No	Large effect	Low
Liampas	2020	General population	Case–control	Linear increase	Migraine adult	12	1074	1961	MD	-0.34	0.15	0.54	NA	72	No	No	NA	No	Low
Liu	2023	General population	Case–control	Linear increase	Parkinson’s disease	71	8543	16,586	SMD	*0.8*	0.61	0.99	< 33%	93.6	No	No	No	Very large effect	Low
Morandi	2021	General population	Case–control	Linear increase	Depression	33			WMD	2.53	1.77	3.3	33–66%	95.4	No	No	Yes	Very large effect	Low
Muntjewerff	2006	General population	Case–control	Linear increase	schizophrenia	8	812	2925	OR	1.7	1.27	2.2	NA	p < 0.05	No	No	NA	No	Low
Guanzhong Ni	2014	Epileptic population	Case–control	Linear increase	Epilepsy treated with VPA	8	266	755	SMD	0.62	0.32	0.92	33–66%	65.6	No	Serious	NA	Large effect	Low
Rabelo	2022	General population	Case–control	Linear increase	ischemic stroke	13	5002	9947	SMD	1.67	1	2.25	NA	99	No	No	Yes	Very large effect	Low
Tian	2023	Stroke-free	Cross-sectional	Linear increase	ICAS	23	1181	5872	SMD	2.25	1.02	3.48	< 33%	90.1	No	No	No	Very large effect	Low
Yan	2022	General population	Case–control	Highest vs lowest category	Obsessive–Compulsive Disorder	3	NA	185	RR	1.13	0.49	1.78	< 33%	74.3	No	Serious	NA	No	Low
Zhang	2018	General population	Case–control	Highest vs lowest category	sclerosis diseases	8	475	740	SMD	1.382	-0.442	3.206	NA	98.7	No	Serious	No	Very large effect	Low
Zhao	2022	General population	Case–control	Highest vs lowest category	Alzheimer disease	30		4788	SMD	0.59	0.36	0.82	< 33%	88.8	No	No	No	Large effect	Low
Boushey	1996	General population	Case–control	Linear increase	Cerebrovacular disease	15		NA	OR	2.5	2	3	NA	p < 0.05	No	No	NA	Large effect	Very low
Chen	2022	PSD patient	Case–control	Linear increase	acute and post stroke depression	11		2789	SMD	0.37	0.07	0.66	< 33%	91.5	No	No	No	No	Very low
Li	2022	General population	Cohort	Linear increase	recurrent stroke	15	2563	23,253	OR	1.6	1.12	1.39	< 33%	87.8	No	No	NA	No	Very low
Liampas	2020	General population	Case–control	Linear increase	Migraine (unspecified age)	3	124	189	MD	0.68	-0.6	1.97	NA	92	No	Serious	NA	No	Very low
Liampas	2020	General population	Case–control	Linear increase	Migraine	16	1243	2240	MD	0.41	0.2	0.61	NA	81	No	No	NA	No	Very low
Morandi	2021	General population	Cohort	Linear increase	Depression	26			RR	1.34	1.19	1.52	33–66%	75.1	No	No	No	No	Very low

Abbreviations:

**Table 7:** Evidence about hyperhomocysteinemia and pregnancy-related outcomes. including evidence about children and adolescents

Author	Year	Type Of Population	Type Of Study	Definition Of Hyperomocisteinemia	Outcome	N° Studies For The Outcome	Number Of Events	Total Sample Size (Outcome)	Type Of Effect Size	Effect Size	95% Ci Low—Outcome	95%Ci High—Outcome	% Studies At High Risk Of Bias	Heterogeneity	Indirectness	Imprecision	Publication Bias	Large Effect	GRADE
Visser	2014	Pregnant women	Case–control	Linear increase	Hypertension	5	NA	NA	MD	0.77	0.27	1.26	NA	48	No	No	No	No	Moderate
Yang	2017	Pregnant women	Case–control	Highest vs lowest category	Neural tube defects	17	1304	3237	SMD	0.06	0.02	0.09	33–66%	0	No	No	No	No	Moderate
Zhang	2022	Pregnant women	Case–control	Highest vs lowest category	Pre-eclampsia	9	4384	26,021	OR	1.33	1.14	1.54	< 33%	71.4	No	No	Yes	No	Moderate
Bala	2021	Pregnant women	Case–control	Linear increase	Early Pregnancy Loss	3		230	OR	1.3	0.68	2.47	NA	0	No	Serious	No	No	Low
Diao	2020	Pregnant women	Case–control	Linear increase	spontaneous abortion	23	2052	3528	SMD	1.34	0.76	1.93	< 33%	88	No	No	No	Very large effect	Low
Gong	2016	Pregnant women	Case–control	Linear increase	DM	11		400	MD	0.77	0.44	1.1	NA	27	No	Serious	No	No	Low
Hogeveen	2012	Pregnant women	Case–control	Linear increase	birth weight	19	3347	21,326	OR	1.4	0.9	1.9	NA	0	No	No	NA	No	Low
Li	2021	Pregnant women	Case–control	Linear increase	recurrent spontaneous abortion	28	??	??	SMD	1	0.72	1.27	NA	94.8	No	No	Yes	Very large effect	Low
Liampas	2020	Children	Case–control	Linear increase	Migraine (children)	1	45	90	MD	0.37	-0.05	0.79	NA	NA	No	Serious	NA	No	Low
Nelen	2000	Pregnant women	Case–control	Linear increase	recurrent early pregnancy loss	10	107	478	OR	2.7	1.4	5.2	NA	No	No	Serious	NA	Large effect	Low
Ulloque-Badaracco	2025	Children and adolescents	Case–control	Linear increase	Obesity	20		7791	SMD	0.77	0.39	1.14	< 33%	86.4	No	No	No	Very large effect	Low
Zheng	2021	Pregnant women	Case–control	Highest vs lowest category	Gestational diabetes mellitus	12		712	SMD	0.55	0.25	0.85	NA	84	No	Serious	No	Large effect	Very low

Abbreviations:

**Table 8:** Evidence about hyperhomocysteinemia and respiratory-related outcomes

Author	Year	Type Of Population	Type Of Study	Definition Of Hyperomocisteinemia	Outcome	N° Studies For The Outcome	Number Of Events	Total Sample Size (Outcome)	Type Of Effect Size	Effect Size	95% Ci Low—Outcome	95%Ci High—Outcome	% Studies At High Risk Of Bias	Heterogeneity	Indirectness	Imprecision	Publication Bias	Large Effect	GRADE
He	2024	General population	Case–control	Linear increase	OSAHS	43			WMD	4.25	2.6	5.91	< 33%	98.7	No	No	No	Very large effect	Low
Xun Niu	2014	General population	Case–control	Linear increase	OSA	10	432	413	RR	3.11	2.08	4.15	NA	71	No	Serious	No	Large effect	Low
Zinellu	2023	General population	Case–control	Highest vs lowest category	COPD	21	678	1139	SMD	0.78	0.48	1.07	NA	79.4	No	No	No	Very large effect	Low

Abbreviations:

**Table 9:** Evidence about hyperhomocysteinemia and other health outcomes of interest

Author	Year	Type Of Population	Type Of Study	Definition Of Hyperomocisteinemia	Outcome	N° Studies For The Outcome	Number Of Events	Total Sample Size (Outcome)	Type Of Effect Size	Effect Size	95% Ci Low—Outcome	95%Ci High—Outcome	% Studies At High Risk Of Bias	Heterogeneity	Indirectness	Imprecision	Publication Bias	Large Effect	GRADE
Chen	2023	General population	Cohort	Linear increase	chronic kidney disease	11	NA	79,416	OR	2.09	1.72	2.55	< 33%	57.7	No	No	No	Large effect	High
Carpenè	2022	COVID-19	Case–control	Linear increase	Favorable prognosis	3	NA	694	SMD	1.75	1.26	2.25	NA	78	No	Serious	NA	Very large effect	Low
Kim	2009	General population	Case–control	Linear increase	psoriasis	16	1172	2091	WMD	3.3	1.58	5.02	< 33%	82.2	No	No	No	Very large effect	Low
Oussalah	2011	General population	Case–control	Linear increase	IBD	28	2370	4342	OR	4.65	3.04	7.09	NA	51	No	No	NA	Very large effect	Low
Sam	2020	General population	Case–control	Linear increase	LES	36	2919	6039	SMD	1.479	1.119	1.839	< 33%	97.3	No	No	Yes	Very large effect	Low
Tsai	2019	General population	Case–control	Linear increase	Vitiligo	22	NA	1448	SMD	0.55	0.262	0.838	< 33%	87.3	No	No	No	Large effect	Low
Tsai	2021	General population	Case–control	Linear increase	Systemic Lupus Erythematosus	50	NA	4396	SMD	1	0.795	1.474	< 33%	97.5	No	No	Yes	Very large effect	Low
Tsai	2019	General population	Case–control	Linear increase	psoriasis	18	965	1893	SMD	0.41	0.21	0.61	< 33%	76.7	No	No	No	No	Low
Verkleij-Hagoort	2007	General population	Case–control	Linear increase	orofacial clefts	2	3341	20,113	OR	2.3	0.4	11.9	NA	67.9	No	No	No	Large effect	Low
Zhong	2019	General population	Case–control	Highest vs lowest category	ulcerative colitis	18	NA	NA	SMD	1.2	0.89	1.51	> 66%	94.5	No	No	No	Very large effect	Low
Li	2021	General population	Case–control	Linear increase	ankylosing spondylitis	9	778	1300	SMD	0.46	0.3	1.23	< 33%	97.3	No	No	No	No	Very low

Abbreviations:

As fully detailed in Supplementary Table 5, using the criteria suggested by the AMSTAR-2, 101 meta-analyses were ranked as critically low, two low, and only one moderate. The most commonly failed critical items were: Item 2 (protocol), Item 7 (excluded studies list), Item 13 (risk of bias interpretation), Item 15 (publication bias), and to a lesser extent Item 9 (RoB assessment quality).

## Main findings

### Cancer-related outcomes

Table 1 shows the association between hyperhomocysteinemia and cancer. Overall, among 5 outcomes analyzed using case–control studies, we found a moderate level of evidence supporting the association between the presence of hyperhomocysteinemia and digestive tract cancers (13 studies; OR = 1.27; 95%CI: 1.15–1.39; I^2^ = 0%). Moreover, again supported by a moderate level of evidence, we found that patients affected by lung cancer reported significantly higher values of homocysteinemia compared to controls (SMD = 0.41; 95%CI: 0.24–0.58; I^2^ = 61.9%). The association between hyperhomocysteinemia and the other cancer-related outcomes was supported by low or very low level of evidence, according to the GRADE, as shown in Table 1.

### Cardiovascular disease-related outcomes

Table 2 shows the association between hyperhomocysteinemia and cardiovascular diseases (CVDs). Several outcomes were supported by a moderate level of evidence according to the GRADE, namely a statistically significant association between high homocysteinemia levels and the presence of aortic aneurysm, retinal artery occlusion, congenital heart defects, and cardiac death in general population. Two associations, i.e., hyperhomocysteinemia and non-fatal myocardial infarction or stenosis, were supported by a moderate level of evidence, but were not statistically significant. All these outcomes were made using case–control studies. The other 26 outcomes were, on the contrary, supported by a low (n = 22) or very low (n = 4) level of evidence.

### Metabolic diseases related outcomes

Table 3 shows the association between hyperhomocysteinemia and metabolic conditions, including fractures, for a total of 21 outcomes. In this category, we found three associations supported by a high level of evidence, meaning that the most common biases analyzed poorly affect these results. In detail, in 28 case–control studies, including 47,704 patients, hyperhomocysteinemia was associated with an increased risk in fracture (HR = 1.25; 95%CI: 1.12–1.40; I^2^ = 45%): these findings were confirmed by the same level of evidence in nine cohort studies (RR = 1.59; 95%CI: 1.30–1.96; I^2^ = 48.5%). Similarly, even if supported by a moderate level of evidence, we found that the presence of a baseline hyperhomocysteinemia was associated with an increased risk of hip fracture (RR = 1.67; 95%CI: 1.17–2.38; I^2^ = 58.2%). Moreover, in nine cohort studies including 5,547 patients affected by diabetes, high homocysteinemia levels were associated with an increased incidence of diabetic retinopathy (RR = 1.93; 95%CI: 1.46–2.53; I^2^ = 26.9%). The other 17 outcomes were supported by a low or very low evidence of evidence.

### Eye diseases related outcomes

As shown in Table 4, four outcomes were analyzed about hyperhomocysteinemia and eye-related conditions, but all supported by a low or very low level of evidence.

### Mortality

Table 5 analyzes the evidence about hyperhomocysteinemia and mortality across different conditions, for a total of five different analyses. The only one supported by a moderate level of evidence was the association between hyperhomocysteinemia and mortality in general population: this analysis was made in 11 studies and 27,737 people with an RR of 1.88 (95%CI: 1.51–2.14; I^2^ = 64.8%). The other associations were supported by a low or very low level of evidence.

### Neurological diseases related outcomes

Table 6 summarizes the evidence about hyperhomocysteinemia and neurological conditions. Overall, 34 outcomes were included in this category. Four outcomes were supported by a high level of evidence. Patients with post-stroke depression (n = 12; SMD = 0.14; 95%CI: 0.05- 0.22; I^2^ = 0%) or intracerebral hemorrhage (n = 7; SMD = 0.59; 95%CI: 0.51–0.68; I^2^ = 0%) reported significantly higher levels of homocysteinemia compared to the controls. Moreover, hyperhomocysteinemia was associated with a higher risk of hemorrhagic (n = 4; OR = 1.99; 95%CI: 1.03–3.84; I^2^ = 32.6%) or ischemic (n = 13; OR = 1.71; 95%CI: 1.39–2.11; I^2^ = 45.2%) stroke in cohort studies. Furthermore, six outcomes were supported by a moderate level of evidence. In particular, we observed that, in cohort studies, hyperhomocysteinemia was associated with an increased risk of any dementia (n = 8; OR = 1.50; 95% CI: 1.13–2; I^2^ = 0%) and of Alzheimer-type dementia (n = 28; RR = 1.15; 95%C: 1.04–1.26; I^2^ = 56.6%) (Table 6).

### Outcomes about pregnancy and children/adolescents

Table 7 shows the evidence on pregnant women and children or adolescents, in 12 outcomes. In three situations, namely pregnant women with hypertension, pre-eclampsia or having newborns with neural tube defects reported significantly higher homocysteinemia levels compared to healthy controls, supported by a moderate level of evidence. All the other nine outcomes were supported by a low level of evidence, except the association with gestational diabetes, supported by a very low level of evidence.

### Respiratory diseases related outcomes

As shown in Table 8, three outcomes were analyzed on hyperhomocysteinemia and respiratory-related conditions, but all were supported by a low level of evidence.

### Other conditions

Finally, eleven outcomes were ranked in this category, containing outcomes not includable in the categories mentioned above, as reported in Table 9. The only one supported by a high level of evidence was the association between hyperhomocysteinemia and the incidence of chronic kidney disease (n = 11 cohort studies; 79,416 free from chronic kidney disease at baseline; OR = 2.09; 95%CI: 1.72–2.55; I^2^ = 57.7%) in which the high heterogeneity detected was counterbalanced by the large effect size found.

## Discussion

This umbrella review integrated an exceptionally large and heterogeneous evidence base, comprising 104 systematic reviews/meta-analyses, covering 126 independent outcomes, and synthesizing data from 2,110 primary studies with nearly 1 million participants (953,793). Across this vast literature, the direction of effect was remarkably consistent: 105/126 outcomes (83.3%) showed a statistically significant association between hyperhomocysteinemia and the outcome of interest. However, the credibility assessment highlights an important nuance: only a minority of associations achieved high or moderate certainty, whereas many signals—despite being statistically significant—were supported by low/very low certainty, reflecting limitations typical of observational evidence (residual confounding, reverse causation, heterogeneity in hyperhomocysteinemia definitions, and selective reporting). Figure 2 summarizes the main clinical findings.

Considering associations supported by high level of evidence, we found an important association between hyperhomocysteinemia and stroke and cerebrovascular phenotypes (ischemic and haemorrhagic stroke; intracerebral haemorrhage). We can hypothesize that hyperhomocysteinemia could be associated with cerebrovascular events through multiple converging pathways. First, hyperhomocysteinemia could promote endothelial dysfunction (reduced nitric oxide bioavailability; impaired vasodilation), fostering vascular reactivity abnormalities and atherogenesis. [15] Moreover, hyperhomocysteinemia is often associated with oxidative stress and vascular inflammation, amplifying lipid oxidation and accelerating plaque development. [16] Third, hyperhomocysteinemia could promote prothrombotic state, including enhanced platelet activation and altered coagulation/fibrinolysis balance, increasing the probability of occlusive events [17]. Intervention evidence is mixed overall, but stroke is one of the clearest areas where lowering homocysteine can translate into benefit in specific contexts. For example, in the CSPPT (China Stroke Primary Prevention Trial), adding folic acid to enalapril significantly reduced first stroke among hypertensive adults in China, a setting with relatively low baseline folate. [18] Moreover, a recent RCT meta-analysis found that folic acid supplementation reduced stroke risk overall, with larger effects in regions without grain folate fortification, consistent with the “baseline deficiency modifies benefit” hypothesis. [19] Taken together, the epidemiology (high-evidence association) plus the pattern of trial results supports a mechanistic model where hyperhomocysteinemia is likely causal or contributory in at least some stroke pathways, but treat-to-lower strategies are most effective when targeted (e.g., high baseline hyperhomocysteinemia, low folate status, primary prevention, or settings without fortification). In this sense, mandatory folate fortification acts as a massive modifier of disease association. In countries with active fortification programs, the population-wide baseline levels of homocysteine are lower, which attenuates the relative risks and "assay sensitivity" of observational studies. Conversely, in non-enriched countries or periods prior to fortification, baseline deficiencies are widespread, making the association between hyperhomocysteinemia and clinical endpoints (such as stroke) much stronger and more distinct.

In our umbrella review, we also found that hyperhomocysteinemia was associated with an increased fracture risk (supported by high certainty), both in case–control and cohort evidence. We believe that several mechanisms can link hyperhomocysteinemia to bone fragility. First, homocysteine can interfere with collagen maturation pathways, finally leading to an impaired collagen cross-linking and deterioration of bone matrix quality, weakening bone independent of bone mineral density. [20] Similarly, hyperhomocysteinemia could promote osteoclastogenesis and suppress osteoblast function, since, as mentioned before, could increase oxidative stress and inflammation. [20] In the B-PROOF trial, vitamin B12 + folic acid lowered homocysteine but did not reduce overall osteoporotic fracture incidence; exploratory analyses suggested possible benefit in very old compliant participants. [21] Furthermore, in our umbrella review, hyperhomocysteinemia was associated with a higher incidence of chronic kidney disease, supported by high certainty. Again, hyperhomocysteinemia may contribute to chronic kidney disease onset/progression via endothelial injury, oxidative stress, and microvascular disease within renal circulation, promoting glomerular and tubulointerstitial damage. [22] Moreover, it is also likely that hyperhomocysteinemia may also reflect systemic metabolic dysregulation (inflammation, insulin resistance, poor nutritional status) that increases CKD susceptibility.

Among the associations supported by moderate level of evidence, we would like to briefly discuss the findings about dementia, also because such findings are supported by cohort studies. Hyperhomocysteinemia, as also mentioned when discussing the findings about stroke, is a risk factor for cerebrovascular contributions (atherosclerosis, small vessel disease, microinfarcts), consistent with mixed vascular-neurodegenerative mechanisms in late-life cognitive decline. Moreover, it was also reported that hyperhomocysteinemia could promote direct neurotoxicity. [23] Finally, experimental data suggest hyperhomocysteinemia-related metabolic stress may facilitate amyloid deposition and tau phosphorylation (mechanistic plausibility, though translating this to human causality remains challenging). [24] In relation to dementia, we should observe that this is one of the most compelling areas where hyperhomocysteinemia lowering may affect meaningful intermediate and clinical endpoints in selected groups. For example, in VITACOG, B-vitamin treatment lowered homocysteine was associated with slower brain atrophy and improved cognitive/clinical outcomes in mild cognitive impairment (especially in those with higher baseline homocysteine). [25] Moreover, neuroimaging work also supports the concept that B vitamins can slow atrophy in brain regions relevant to Alzheimer’s pathology. [26] These findings align with the epidemiology and provide a biologically coherent signal that reversal of hyperhomocysteinemia may be beneficial in early cognitive impairment, though definitive dementia-prevention trials remain a priority.

Finally, our work shows that hyperhomocysteinemia is associated with a significant 1.88-fold increased risk of mortality in the general population, likely serving as an integrated biomarker of systemic vascular damage, renal decline, and cellular aging. [27] Similarly, our umbrella review supports the notion that hyperhomocysteinemia to digestive tract and lung cancers: other than the reasons mentioned before, we can also consider how disruptions in the one-carbon metabolic pathway alter DNA methylation, provoke DNA strand breaks, and promote oncogenic transformations. [28].

Despite the large number of studies included, several limitations should be acknowledged. First, the most important, included meta-analyses were derived from observational studies; therefore, residual confounding (renal function, nutritional status, inflammation, socioeconomic factors, comorbidities) and reverse causality remain key threats to causal inference. Second, from a clinical perspective, a high heterogeneity in exposure definition was observed: hyperhomocysteinemia thresholds and exposure contrasts varied across meta-analyses (highest vs lowest category, different cutoffs, or linear increments), potentially inflating heterogeneity and limiting comparability. Third, while statistical significance is highly pervasive (83.3% of outcomes), true clinical credibility should be considered only for high- and moderate-certainty clusters, therefore for the outcomes in which the presence of biases is limited. Fourth, methodological quality of included reviews: several meta-analyses were rated as low/critically low quality by AMSTAR-2 criteria (e.g., absent a priori protocol, incomplete reporting, limited publication-bias assessment), which may bias pooled estimates.

In conclusion, across a very large literature base, hyperhomocysteinemia showed a pervasive association with adverse health outcomes, with 83.3% of outcomes indicating significant relationships. Associations supported by high or moderate certainty clustered around clinically meaningful endpoints—particularly stroke, fractures/hip fracture, incident chronic kidney disease, dementia/Alzheimer-type dementia, general-population mortality, selected cardiovascular phenotypes, and major pregnancy complications. Mechanistically, these links are consistent with hyperhomocysteinemia -driven endothelial dysfunction, oxidative stress, inflammation, thrombosis, and one-carbon metabolism disruption. Importantly, the intervention literature suggests that lowering homocysteine can improve outcomes in selected contexts, most consistently for stroke prevention in low-folate settings and for neurodegeneration-related intermediate/clinical outcomes in mild cognitive impairment, while benefits are less consistent in advanced disease states. Future research should prioritize well-designed trials and pragmatic strategies that target high-risk, biologically enriched groups (e.g., elevated hyperhomocysteinemia with folate/B12 deficiency, no folate fortification, early disease stages), while integrating renal function and nutritional assessment to distinguish when hyperhomocysteinemia is a causal factor versus a severity marker.

## Supplementary Information

Below is the link to the electronic supplementary material.

Supplementary file1 (DOCX 163 KB)

## Data Availability

No datasets were generated or analysed during the current study.
