# Supplementary material for: Hyperhomocysteinemia, a risk factor for various health conditions: an umbrella review of systematic reviews with meta-analysis
Source: Aging Clin Exp Res. 2026 Jul 10;38(1):183. doi: 10.1007/s40520-026-03433-0 (PMC13550087; doi:10.1007/s40520-026-03433-0)
Supplement: Supplementary file 1 [file 40520_2026_3433_MOESM1_ESM.docx]

**Supplementary Material 1. Search strategy, by database**

| **Database** | **Search strategy** | **#** |
| --- | --- | --- |
| **Pubmed** | ("hyperhomocysteinemia"[MeSH Terms] OR "hyperhomocysteinemia"[All Fields] OR "hyperhomocysteinemias"[All Fields] OR "homocysteinaemia"[All Fields] OR "homocysteinemia"[Supplementary Concept] OR "homocysteinemia"[All Fields] OR "homocysteinemia"[All Fields] OR "hyper-homocysteinaemia"[All Fields] OR "hyper-homocysteinemia"[All Fields] OR "hyperhomocysteinaemia"[All Fields] OR "hyperhomocysteinemia"[All Fields] OR "homocystein"[All Fields] OR "homocysteine"[MeSH Terms] OR "homocysteine"[All Fields] OR "homocysteine s"[All Fields] OR "homocysteines"[All Fields]) AND (meta-analysis[Filter] OR systematicreview[Filter]) | **642** |
| **Embase** | (('hyperhomocysteinemia'/exp OR 'homocysteinaemia' OR 'homocysteinemia' OR 'hyper-homocysteinaemia' OR 'hyper-homocysteinemia' OR 'hyperhomocysteinaemia' OR 'hyperhomocysteinemia') OR ('homocysteine'/exp OR '2 amino 4 mercaptobutyric acid' OR 'betahomocystein' OR 'dextro levo homocysteine' OR 'dl homocysteine' OR 'homocysteine' OR 'l homocysteine' OR 'levo homocysteine')) AND ('meta analysis'/exp OR 'meta-analysis' OR ‘systematic review’/exp) | **1278** |
| **WoS** | TS=("hyperhomocysteinemia" OR "hyperhomocysteinemias" OR "homocysteinaemia" OR "homocysteinemia" OR "hyper-homocysteinaemia" OR "hyper-homocysteinemia" OR "homocystein" OR "homocysteine" OR "homocysteine s" OR "homocysteines") AND TS=("meta-analysis" OR "systematic review") | **1247** |

**Supplementary Table 2. Criteria evidence for the GRADE of the outcomes included.**

| **Downgrade** | **Risk of bias** | **Inconsistency** | **Indirectness** | **Imprecision** | **Publication bias** | **Large effect** |
| --- | --- | --- | --- | --- | --- | --- |
| **-1** | Presence of 33-66% of studies with a high risk of bias according to criteria included in the systematic review or risk of bias not available in the text | I^2^ 50-74% or p<0.05 or not available | The question being addressed by the guideline panel is different from the available evidence regarding the PICO | The overall number of individuals included in studies is low (less than 1000 individuals) OR (b) large 95% confidence intervals. | Egger’s test (p-value) <0.05 or evidence of publication bias or not available | RR/OR/HR between 2 and 3 or SMD between 0.5 and 0.7 (absolute value) (+1) |
| **-2** | Presence >66% of studies with a high risk of bias according to criteria included in the systematic review | I^2^ ≥ 75% | The question being addressed by the guideline panel is markedly different from the available evidence regarding the PICO | The overall number of individuals included in studies is low (less than 1000 individuals AND (b) large 95% confidence intervals. | - | RR/OR/HR  > 3 or SMD >0.7 (absolute value) (+2) |

**Abbreviations: OR:** Odds ratio**; PICO:** Population, Intervention, Comparison and Outcomes; **RR**: Risk Ratio; **HR**: Hazard Ratio

**Supplementary Table 3. List of the excluded references**

| **Study** | **Title** | **Published Year** | **Reason** |
| --- | --- | --- | --- |
|  | Dose-dependent effects of folic acid on blood concentrations of homocysteine: a meta-analysis of the randomized trials | 2005 | Wrong study design |
| Akbari 2024 | Impact of Statin or Fibrate Therapy on Homocysteine Concentrations: A Systematic Review and Meta-analysis | 2024 | full-text not available |
| Akdas 2024 | Assessment of the Relationship between Amino Acid Status and Parkinson's Disease: A Comprehensive Review and Meta-analysis | 2024 | No data about hyperhomocysteinemia |
| Almeida 2008 | Homocysteine and depression in later life | 2008 | full-text not available |
| Ames 2022 | Relevance of Plasma Homocysteine and Methylenetetrahydrofolate Reductase 677TT Genotype in Sickle Cell Disease: A Systematic Review and Meta-Analysis | 2022 | Wrong study design |
| Ammouri 2017 | Venous thromboembolism and hyperhomocysteinemia as first manifestation of pernicious anemia: A case series | 2017 | No data about hyperhomocysteinemia |
| Amorim 2013 | MTRR 66A>G polymorphism as maternal risk factor for Down syndrome: a meta-analysis | 2013 | no data about hyperhomocisteinemia |
| Ansari 2016 | Homocysteine and mild cognitive impairment: Are these the tools for early intervention in the dementia spectrum? | 2016 | No data about hyperhomocysteinemia |
| Arentz 2017 | Nutritional supplements and herbal medicines for women with polycystic ovary syndrome; a systematic review and meta-analysis | 2017 | Wrong study design |
| Arhip 2024 | Late-onset methylmalonic acidemia and homocysteinemia (cblC disease): systematic review | 2024 | no full text |
| Asbaghi 2021 | Folic Acid Supplementation Improves Glycemic Control for Diabetes Prevention and Management: A Systematic Review and Dose-Response Meta-Analysis of Randomized Controlled Trials | 2021 | Wrong study design |
| Bahtiri 2015 | Relationship of homocysteine levels with lumbar spine and femur neck BMD in postmenopausal women | 2015 | full-text not available |
| Balandeh 2021 | Serum Vitamins and Homocysteine Levels in Obsessive-Compulsive Disorder: A Systematic Review and Meta-Analysis | 2021 | full-text not available |
| Batista 2022 | The role of vitamin B12 in viral infections: a comprehensive review of its relationship with the muscle-gut-brain axis and implications for SARS-CoV-2 infection | 2022 | No data about hyperhomocysteinemia |
| Behere 2021 | Maternal Vitamin B12 Status During Pregnancy and Its Association With Outcomes of Pregnancy and Health of the Offspring: A Systematic Review and Implications for Policy in India | 2021 | No data about hyperhomocysteinemia |
| Behrens 2020 | Vitamin B-Can it prevent cognitive decline? A systematic review and meta-analysis | 2020 | No data about hyperhomocysteinemia |
| Bellos 2020 | Markers of endothelial dysfunction and arterial stiffness in patients with early-stage autosomal dominant polycystic kidney disease: a meta-analysis | 2020 | No data about hyperhomocysteinemia |
| Bellos 2021 | Markers of endothelial dysfunction and arterial stiffness in patients with early-stage autosomal dominant polycystic kidney disease: A meta-analysis | 2021 | Wrong study design |
| Bennett 2014 | MTHFR C677T genotype is not associated with ischaemic heart disease and stroke in a large prospective study in china | 2014 | No data about hyperhomocysteinemia |
| Bergmann 2013 | Protein Z deficiency and Lipoprotein (a) increase are the most frequent abnormalities in women with recurrent miscarriage | 2013 | No data about hyperhomocysteinemia |
| Beydoun 2014 | Epidemiologic studies of modifiable factors associated with cognition and dementia: systematic review and meta-analysis | 2014 | doubled |
| Blanco 2016 | Maternal biomarkers of methylation status and non-syndromic orofacial cleft risk: a meta-analysis | 2016 | No data about hyperhomocysteinemia |
| Bleys 2006 | Vitamin-mineral supplementation and the progression of atherosclerosis: a meta-analysis of randomized controlled trials | 2006 | Wrong study design |
| Blom 2009 | Folic Acid, Methylation and Neural Tube Closure in Humans | 2009 | No data about hyperhomocysteinemia |
| Bonnot 2014 | Diagnostic and treatment implications of psychosis secondary to treatable metabolic disorders in adults: a systematic review | 2014 | No data about hyperhomocysteinemia |
| Booth 2000 | Preventive health care, 2000 update: screening and management of hyperhomocysteinemia for the prevention of coronary artery disease events. The Canadian Task Force on Preventive Health Care | 2000 | No data about hyperhomocysteinemia |
| Bozzatello 2024 | Metabolic Dysfunctions, Dysregulation of the Autonomic Nervous System, and Echocardiographic Parameters in Borderline Personality Disorder: A Narrative Review | 2024 | No data about hyperhomocysteinemia |
| Brattström 1998 | Common methylenetetrahydrofolate reductase gene mutation leads to hyperhomocysteinemia but not to vascular disease: the result of a meta-analysis | 1998 | No data about hyperhomocysteinemia |
| Bromberg 2008 | No association between global leukocyte DNA methylation and homocysteine levels in schizophrenia patients | 2008 | No data about hyperhomocysteinemia |
| Brumat 2019 | Preliminary results from a new GWAS meta-analysis point at new loci for age-related hearing loss (ARHL) | 2019 | conference abstract |
| Butler 2006 | Oral vitamin B12 versus intramuscular vitamin B12 for vitamin B12 deficiency: a systematic review of randomized controlled trials | 2006 | Wrong study design |
| Cacione 2016 | Pharmacological treatment for Buerger's disease | 2016 | wrong study design |
| Cahill 2003 | Meta-analysis of plasma homocysteine, serum folate, serum vitamin B(12), and thermolabile MTHFR genotype as risk factors for retinal vascular occlusive disease | 2003 | No data about hyperhomocysteinemia |
| Cai 2022 | Vitamins and <i>Helicobacter pylori</i>: An Updated Comprehensive Meta-Analysis and Systematic Review | 2022 | No data about hyperhomocysteinemia |
| Cao 2013 | Association study between methylenetetrahydrofolate reductase polymorphisms and unexplained recurrent pregnancy loss: A meta-analysis | 2013 | No data about hyperhomocysteinemia |
| Cao 2023 | Meta-analysis of the clinical efficacy of compound α -ketoacid tablets combined with low-protein diet in diabetic kidney disease | 2023 | Wrong study design |
| Carlus 2016 | Is MTHFR 677 C>T polymorphism clinically important in Polycystic Ovarian Syndrome (PCOS)? A case-control study, meta-analysis and trial sequential analysis | 2016 | No data about hyperhomocysteinemia |
| Casas 2005 | Homocysteine and stroke: evidence on a causal link from mendelian randomisation | 2005 | No data about hyperhomocysteinemia |
| Çetintas 2014 | Association between polymorphism of <i>MTHFR</i> c.677C&gt;T and risk of cardiovascular disease in Turkish population: a meta-analysis for 2.780 cases and 3.022 controls | 2014 | No data about hyperhomocysteinemia |
| Chapman 2016 | Association between metformin and vitamin B<sub>12</sub> deficiency in patients with type 2 diabetes: A systematic review and meta-analysis | 2016 | Wrong study design |
| Chaudhary 2019 | Serum Homocysteine Could Be Used as a Predictive Marker for Chronic Obstructive Pulmonary Disease: A Meta-Analysis | 2019 | doubled |
| Chauhan 2012 | Common variants of homocysteine metabolism pathway genes and risk of type 2 diabetes and related traits in Indians | 2012 | No data about hyperhomocysteinemia |
| Chen 2009 | C677T methylenetetrahydrofolate reductase gene polymorphisms in bipolar disorder: An association study in the Chinese population and a meta-analysis of genetic association studies | 2009 | Wrong study design |
| Chen 2020 | Dietary Intake of Homocysteine Metabolism-Related B-Vitamins and the Risk of Stroke: A Dose-Response Meta-Analysis of Prospective Studies | 2020 | No data about hyperhomocysteinemia |
| Chen 2021 | Oxidative stress marker aberrations in children with autism spectrum disorder: a systematic review and meta-analysis of 87 studies (N = 9109) | 2021 | No data about hyperhomocysteinemia |
| Clarke 2003 | Homocysteine, renal function, and risk of cardiovascular disease | 2003 | Wrong study design |
| Clarke 2008 | B-vitamins and prevention of dementia | 2008 | No data about hyperhomocysteinemia |
| Clarke 2009 | Folic acid, homocysteine and risk of coronary heart disease and stroke: meta-analysis of 8 trials involving 37,500 participants | 2009 | conference abstract |
| Clarke 2012 | Homocysteine and Coronary Heart Disease: Meta-analysis of <i>MTHFR</i> Case-Control Studies, Avoiding Publication Bias | 2012 | No data about hyperhomocysteinemia |
| Clarke 2012 | Homocysteine and coronary heart disease: meta-analysis of MTHFR case-control studies, avoiding publication bias | 2012 | Wrong study design |
| Clarke 2014 | Effects of homocysteine lowering with B vitamins on cognitive aging: meta-analysis of 11 trials with cognitive data on 22,000 individuals | 2014 | Wrong study design |
| Collin 2010 | Circulating Folate, Vitamin B<sub>12</sub>, Homocysteine, Vitamin B<sub>12</sub> Transport Proteins, and Risk of Prostate Cancer: a Case-Control Study, Systematic Review, and Meta-analysis | 2010 | doubled |
| Cong 2021 | Diagnostic utility of fluid biomarkers in multiple system atrophy: a systematic review and meta-analysis | 2021 | No data about hyperhomocysteinemia |
| Coppedè 2014 | The MTRR 66A>G polymorphism and maternal risk of birth of a child with Down syndrome in Caucasian women: A case-control study and a meta-analysis | 2014 | No data about hyperhomocysteinemia |
| Cornelis 2015 | Genome-wide association study of selenium concentrations | 2015 | No data about hyperhomocysteinemia |
| Cotlarciuc 2014 | Effect of genetic variants associated with plasma homocysteine levels on stroke risk | 2014 | No data about hyperhomocysteinemia |
| Cronin 2005 | Dose-related association of MTHFR 677T allele with risk of ischemic stroke: evidence from a cumulative meta-analysis | 2005 | No data about hyperhomocysteinemia |
| Cui 2016 | MTHFR C677T mutation increased the risk of Ischemic Stroke, especially in large-artery atherosclerosis in adults: an updated meta-analysis from 38 researches | 2016 | No data about hyperhomocysteinemia |
| Czapski 2012 | Association between plasma biomarkers, CDK5 polymorphism and the risk of Alzheimer's disease | 2012 | No data about hyperhomocysteinemia |
| Dai 2016 | Association of homocysteine level with biopsy-proven non-alcoholic fatty liver disease: A meta-analysis | 2016 | doubled |
| Danesh 2000 | Low grade inflammation and coronary heart disease: prospective study and updated meta-analyses | 2000 | No data about hyperhomocysteinemia |
| Danesh 2000 | Chlamydia pneumoniae IgG titres and coronary heart disease: prospective study and meta-analysis | 2000 | no full text |
| Dardiotis 2017 | Vitamin B12, folate, and homocysteine levels and multiple sclerosis: A meta-analysis | 2017 | doubled |
| Dave 2018 | EVALUATION OF THE NEUROVASCULAR HYPOTHESES OF ALZHEIMER'S DISEASE (AD): META-ANALYSIS OF GLIAL, NEURONAL AND VASCULAR BIOMARKERS | 2018 | conference abstract |
| Debreceni 2014 | The role of homocysteine-lowering B-vitamins in the primary prevention of cardiovascular disease | 2014 | No data about hyperhomocysteinemia |
| Deminice 2016 | Creatine supplementation decreased homocysteine plasma levels in rats but not humans: A critical review with meta-analysis | 2016 | Wrong study design |
| Deng 2023 | Risk of Anemia and Vitamin B12 Deficiency in Patients with Type 2 Diabetes Mellitus on Metformin Therapy: A Systematic Review and Meta-Analysis | 2023 | No data about hyperhomocysteinemia |
| DenHeijer 2005 | Homocysteine, MTHFR and risk of venous thrombosis: a meta-analysis of published epidemiological studies | 2005 | No data about hyperhomocysteinemia |
| DenHeijer 2005 | Homocysteine, <i>MTHFR</i> and risk of venous thrombosis:: a meta-analysis of published epidemiological studies | 2005 | No data about hyperhomocysteinemia |
| deOliveiraLeite 2021 | Homocysteine levels and cardiovascular risk factors in children and adolescents: systematic review and meta-analysis | 2021 | Doubled |
| De-Regil 2015 | Effects and safety of periconceptional oral folate supplementation for preventing birth defects | 2015 | Wrong study design |
| Díaz 2023 | Effect of B-vitamins supplementation on cognitive decline in patients with Alzheimer's disease. A systematic review and meta-analysis | 2023 | No data about hyperhomocysteinemia |
| Dong 2020 | Plasma homocysteine, folate and vitamin B12 levels in Parkinson's disease in China: A meta-analysis | 2020 | doubled |
| Du 2019 | Polymorphisms of methalenetetrahydrofolate reductase in recurrent pregnancy loss: an overview of systematic reviews and meta-analyses | 2019 | No data about hyperhomocysteinemia |
| Duan 2018 | Novel susceptibility loci for moyamoya disease revealed by a genome-wide association study | 2018 | full-text not available |
| Duffy 2014 | Biomarker responses to folic acid intervention in healthy adults: A meta-analysis of randomized controlled trials | 2014 | No data about hyperhomocysteinemia |
| Ebbing 2010 | Combined analyses and extended follow-up of two randomized controlled homocysteine-lowering B-vitamin trials | 2010 | No data about hyperhomocysteinemia |
| Érszegi 2024 | Not first-line antihypertensive agents, but still effective—The efficacy and safety of imidazoline receptor agonists: A network meta-analysis | 2024 | Wrong study design |
| Esmaiel 2023 | Association between <i>MTHFR</i> C677T variant and risk for congenital heart defects in Egyptian children: a case-control study including meta-analysis based on 147 cases and 143 controls | 2023 | No data about hyperhomocysteinemia |
| Fabiani 2019 | Dietary Patterns in Relation to Low Bone Mineral Density and Fracture Risk: A Systematic Review and Meta-Analysis | 2019 | No data about hyperhomocysteinemia |
| Fallah 2024 | Folate Biomarkers, Folate Intake, and Risk of Death From All Causes, Cardiovascular Disease, and Cancer: A Systematic Review and Dose-Response Meta-Analysis of Prospective Cohort Studies | 2024 | No data about hyperhomocysteinemia |
| Fan 2021 | Efficacy and safety of Xinkeshu in the treatment of angina pectoris of coronary heart disease: A protocol for systematic review and meta-analysis | 2021 | Wrong study design |
| Fan 2022 | Methylenetetrahydrofolate Reductase Gene rs1801133 and rs1801131 Polymorphisms and Essential Hypertension Risk: A Comprehensive Analysis | 2022 | No data about hyperhomocysteinemia |
| Fang 2014 | Methionine synthase reductase A66G polymorphism and leukemia risk: Evidence from published studies | 2014 | No data about hyperhomocysteinemia |
| Fang 2021 | Retinal vein occlusion and chronic kidney disease: A meta-analysis | 2021 | full-text not available |
| Fanning 2014 | The epidemiology of silent brain infarction: a systematic review of population-based cohorts | 2014 | No data about hyperhomocysteinemia |
| Fatahi 2019 | Effects of folic acid supplementation on C-reactive protein: A systematic review and meta-analysis of randomized controlled trials | 2019 | Wrong study design |
| Fernandes 2024 | Hyperhomocysteinemia, Vitamin B12 and Folate Deficiency: The Relationship With Obesity And Cardiovascular Risk | 2024 | No data about hyperhomocysteinemia |
| FloresTorres 2022 | Long-term dietary and supplemental intake of folate, vitamin B6 and vitamin B12 and the incidence of Parkinson's disease in a sample of US women and men | 2022 | No data about hyperhomocysteinemia |
| Ford 2012 | Effect of homocysteine lowering treatment on cognitive function: A systematic review and meta-analysis of randomized controlled trials | 2012 | Wrong study design |
| Ford 2019 | Effect of Vitamin B Supplementation on Cognitive Function in the Elderly: A Systematic Review and Meta-Analysis | 2019 | No data about hyperhomocysteinemia |
| Fraguas 2019 | Oxidative Stress and Inflammation in First-Episode Psychosis: A Systematic Review and Meta-analysis (Publication with Expression of Concern) | 2019 | No data about hyperhomocysteinemia |
| Frustaci 2012 | Oxidative stress-related biomarkers in autism: Systematic review and meta-analyses | 2012 | No data about hyperhomocysteinemia |
| Fu 2014 | Association of methylenetetrahydrofolate reductase gene C677T polymorphism with polycystic ovary syndrome risk: a systematic review and meta-analysis update | 2014 | Wrong study design |
| Fu 2019 | Evidence on the causal link between homocysteine and hypertension from a meta-analysis of 40 173 individuals implementing Mendelian randomization | 2019 | Wrong study design |
| Gadidala 2023 | Effect of garlic extract on markers of lipid metabolism and inflammation in coronary artery disease (CAD) patients: A systematic review and meta-analysis | 2023 | Wrong study design |
| Gaiday 2018 | Effect of homocysteine on pregnancy: A systematic review | 2018 | Wrong study design |
| Gao 2021 | Livedoid vasculopathy and its association with genetic variants: A systematic review | 2021 | No data about hyperhomocysteinemia |
| Gaysina 2008 | No association with the 5,10-methylenetetrahydrofolate reductase gene and major depressive disorder: Results of the depression case control (DeCC) study and a meta-analysis | 2008 | No data about hyperhomocysteinemia |
| Ghoreshi 2024 | Effect of vitamin D supplementation on clinical outcomes in adult patients with COVID-19: A GRADE-assessed systematic review and meta-analysis of randomized controlled trials | 2024 | Wrong study design |
| Gillies 2021 | Exploring trajectories in dietary adequacy of the B vitamins folate, riboflavin, vitamins B(6) and B(12), with advancing older age: a systematic review | 2021 | Wrong study design |
| Giri 2023 | Prothrombotic States in Portal Vein Thrombosis and Budd-Chiari Syndrome in India: A Systematic Review and Meta-Analysis | 2023 | conference abstract |
| Giri 2023 | Prothrombotic states in portal vein thrombosis and Budd-Chiari syndrome in India: A systematic review and meta-analysis | 2023 | No data about hyperhomocysteinemia |
| Gluszek 2021 | The significance of homocysteine in patients with hypertension | 2021 | No data about hyperhomocysteinemia |
| Gofir 2021 | Folic Acid Treatment for Patients with Vascular Cognitive Impairment: A Systematic Review and Meta-Analysis | 2021 | No data about hyperhomocysteinemia |
| Gofir 2022 | Folic Acid Treatment for Patients with Vascular Cognitive Impairment: A Systematic Review and Meta-Analysis | 2022 | No data about hyperhomocysteinemia |
| Gorjipour 2013 | Serum level of homocysteine, folate and vitamin-B12 in epileptic patients under carbamazepine and sodium valproate treatment: a systematic review and meta-analysis | 2013 | No data about hyperhomocysteinemia |
| Goualou 2023 | Venous Thromboembolism Associated with Assisted Reproductive Technology: A Systematic Review and Meta-analysis | 2023 | No data about hyperhomocysteinemia |
| Goulis 2017 | PCOS and cardiovascular disease | 2017 | No data about hyperhomocysteinemia |
| Goyal 2017 | Using CSF markers to validate the vascular hypotheses of Alzheimer's disease (AD): A metaanalysis | 2017 | conference abstract |
| Günther 2020 | Simultaneous appearance of idiopathic Parkinson's syndrome (IPS) and osteoporosis (OPO): Temporary results of the schwarzachgera survey | 2020 | No data about hyperhomocysteinemia |
| Guo 2016 | Serum homocysteine levels and diabetic neuropathy in patients with type 2 diabetes mellitus: A systematic review and meta-analysis | 2016 | doubled |
| Guo 2021 | Shexiang Baoxin Pill for Acute Myocardial Infarction: Clinical Evidence and Molecular Mechanism of Antioxidative Stress | 2021 | Wrong study design |
| Guzelant 2018 | Hypercoagulability as a cause of thrombosis in Behçet's syndrome: A systematic review and meta analysis | 2018 | No data about hyperhomocysteinemia |
| Guzelant 2019 | Hypercoagulability as a cause of thrombosis in behÇet's syndrome: A systematic review and meta-analysis | 2019 | No data about hyperhomocysteinemia |
| GuzelantOzkose 2022 | FACTORS ASSOCIATED with THROMBOSIS in BEHÇET SYNDROME: A SYSTEMATIC REVIEW and META-ANALYSIS | 2022 | No data about hyperhomocysteinemia |
| Hankey 2012 | Antiplatelet therapy offset the benefits of lowering homocysteine in patients with previous stroke or transient ischemic attack (TIA). Results of a randomised, placebo-controlled, trial | 2012 | No data about hyperhomocysteinemia |
| Hankey 2012 | B vitamin supplementation and incidence of cancer in patients with previous stroke or transient ischemic attack (TIA): Results of a randomised trial | 2012 | No data about hyperhomocysteinemia |
| Hankey 2013 | Effect of B vitamins and lowering homocysteine on cognitive impairment in patients with previous stroke or transient ischemic attack: A prespecified secondary analysis of a randomized, placebo-controlled trial and meta-analysis | 2013 | No data about hyperhomocysteinemia |
| Harrison 2017 | Predicting Risk of Cognitive Decline in Very Old Adults Using Three Models: The Framingham Stroke Risk Profile; the Cardiovascular Risk Factors, Aging, and Dementia Model; and Oxi-Inflammatory Biomarkers | 2017 | Wrong study design |
| Hasan 2012 | Towards the identification of blood biomarkers for acute stroke in humans: A comprehensive systematic review | 2012 | No data about hyperhomocysteinemia |
| Haywood 2005 | Thrombophilia and first arterial ischaemic stroke: a systematic review | 2005 | No data about hyperhomocysteinemia |
| He 2014 | Homocysteine level and risk of different stroke types: A meta-analysis of prospective observational studies | 2014 | doubled |
| Herrmann 2006 | Significance of hyperhomocysteinemia | 2006 | Wrong study design |
| Herrmann 2007 | The role of hyperhomocysteinemia as well as folate, vitamin B(6) and B(12) deficiencies in osteoporosis: a systematic review | 2007 | No data about hyperhomocysteinemia |
| Hogeveen 2012 | Maternal homocysteine and small-for-gestational-age offspring: Systematic review and meta-analysis | 2012 | doubled |
| Holmen 2021 | Hyperhomocysteinemia and Ischemic Stroke: A Potential Dose-Response Association-A Systematic Review and Meta-analysis | 2021 | doubled |
| Holmes 2011 | Effect modification by population dietary folate on the association between MTHFR genotype, homocysteine, and stroke risk: a meta-analysis of genetic studies and randomised trials | 2011 | No data about hyperhomocysteinemia |
| Holmes 2011 | Effect modification by population dietary folate on the association between <i>MTHFR</i> genotype, homocysteine, and stroke risk: a meta-analysis of genetic studies and randomised trials | 2011 | No data about hyperhomocysteinemia |
| Hou 2015 | Genetic polymorphism of MTHFR C677T and premature coronary artery disease susceptibility: A meta-analysis | 2015 | No data about hyperhomocysteinemia |
| Hu 2013 | Elevated homocysteine levels in levodopa-treated idiopathic Parkinson's disease: A meta-analysis | 2013 | No data about hyperhomocysteinemia |
| Hu 2016 | Homocysteine and Alzheimer's Disease: Evidence for a Causal Link from Mendelian Randomization | 2016 | doubled |
| Huang 2011 | High consumption of Ω-3 polyunsaturated fatty acids decrease plasma homocysteine: a meta-analysis of randomized, placebo-controlled trials | 2011 | No data about hyperhomocysteinemia |
| Huang 2012 | Meta-analysis of B vitamin supplementation on plasma homocysteine, cardiovascular and all-cause mortality | 2012 | No data about hyperhomocysteinemia |
| Huang 2020 | The Prognostic Value of Homocysteine in Acute Ischemic Stroke Patients: A Systematic Review and Meta-Analysis | 2020 | No data about hyperhomocysteinemia |
| Huang 2021 | The Prognostic Value of Homocysteine in Acute Ischemic Stroke Patients: A Systematic Review and Meta-Analysis | 2021 | No data about hyperhomocysteinemia |
| Humphrey 2008 | Homocysteine level and coronary heart disease incidence: a systematic review and meta-analysis | 2008 | doubled |
| Jaiswal 2024 | C-Reactive Protein, and Homocysteine as a Predictors of Post Stroke Depression Among Stroke Patients: A Meta-Analysis and Meta Regression | 2024 | doubled |
| Janssen 2005 | Retinal vein occlusion: a form of venous thrombosis or a complication of atherosclerosis? A meta-analysis of thrombophilic factors | 2005 | No data about hyperhomocysteinemia |
| Ji 2019 | Modifiable risk factors for carotid atherosclerosis: A meta-analysis and systematic review | 2019 | No data about hyperhomocysteinemia |
| Jiang 2025 | Potential Biomarkers of Post-stroke Cognitive Impairment in Chinese Population: a Systematic Review and Meta-Analysis | 2025 | No data about hyperhomocysteinemia |
| Jin 2021 | Elevated homocysteine levels in patients with heart failure: A systematic review and meta-analysis | 2021 | doubled |
| Jin 2022 | Continuous supplementation of folic acid in pregnancy and the risk of perinatal depression-A meta-analysis | 2022 | No data about hyperhomocysteinemia |
| Kai 2014 | Plasma homocysteine levels in patients with subclinical hypothyroidism: A meta-analysis | 2014 | conference abstract |
| Kang 2014 | Association of the A1298C polymorphism in MTHFR gene with ischemic stroke | 2014 | No data about hyperhomocysteinemia |
| Karimian 2016 | Methionine synthase A2756G transition might be a risk factor for male infertility: Evidences from seven case-control studies | 2016 | No data about hyperhomocysteinemia |
| Kataria 2021 | Effect of Vitamin B6, B9, and B12 Supplementation on Homocysteine Level and Cardiovascular Outcomes in Stroke Patients: A Meta-Analysis of Randomized Controlled Trials | 2021 | No data about hyperhomocysteinemia |
| Keijzer 2007 | No interaction between factor V Leiden and hyperhomocystein-emia or MTHFR 677TT genotype in venous thrombosis - Results of a meta-analysis of published studies and a large case-only study | 2007 | No data about hyperhomocysteinemia |
| Kelly 2002 | Homocysteine, MTHFR 677C-->T polymorphism, and risk of ischemic stroke: results of a meta-analysis | 2002 | No data about hyperhomocysteinemia |
| Kenet 2010 | Impact of Thrombophilia on Risk of Arterial Ischemic Stroke or Cerebral Sinovenous Thrombosis in Neonates and Children A Systematic Review and Meta-Analysis of Observational Studies | 2010 | Doubleds |
| Khan 2022 | HYPERHOMOCYSTEINEMIA POTENTIATING HYPERCOAGULABILITY LEADING TO RARE TRIFECTA OF DVT, PULMONARY EMBOLISM, AND LEFT VENTRICULAR THROMBUS FORMATION | 2022 | conference abstract |
| Khoja 2023 | Risk Factors for Premature Coronary Heart Disease in Women Compared to Men: Systematic Review and Meta-Analysis | 2023 | full-text not available |
| Kielstein 2008 | Two cardiovascular risk factors in one? Homocysteine and its relation to glomerular filtration rate | 2008 | No data about hyperhomocysteinemia |
| Kielstein 2008 | Two cardiovascular risk factors in one? Homocysteine and its relation to glomerular filtration rate. A meta-analysis of 41 studies with 27,000 participants | 2008 | Doubleds |
| Kim 2003 | Association between factor V Leiden, prothrombin G20210A, and methylenetetrahydrofolate reductase C677T mutations and events of the arterial circulatory system: a meta-analysis of published studies | 2003 | No data about hyperhomocysteinemia |
| Kim 2020 | Homocysteine, folic acid, and vitamin B12 levels in patients on isotretinoin therapy for acne vulgaris: A meta-analysis | 2020 | No data about hyperhomocysteinemia |
| Kim 2022 | Potential Biomarkers for Post-Stroke Cognitive Impairment: A Systematic Review and Meta-Analysis | 2022 | No data about hyperhomocysteinemia |
| Kirsty 2022 | The Relationship of Cobalamin and/or Folate to the Patient-Centred Outcomes in Multiple Sclerosis: A Systematic Review and Meta-analysis | 2022 | No data about hyperhomocysteinemia |
| Kjaergaard 2022 | Homocysteine and female fertility, pregnancy loss and offspring birthweight: a two-sample Mendelian randomization study | 2022 | No data about hyperhomocysteinemia |
| Klerk 2002 | MTHFR 677C-->T polymorphism and risk of coronary heart disease: a meta-analysis | 2002 | No data about hyperhomocysteinemia |
| Kluijtmans 1997 | Thermolabile methylenetetrahydrofolate reductase in coronary artery disease | 1997 | No data about hyperhomocysteinemia |
| Kluijtmans 2001 | Methylenetetrahydrofolate reductase genotypes and predisposition to atherothrombotic disease - Evidence that all three MTHFR C677T genotypes confer different levels of risk | 2001 | No data about hyperhomocysteinemia |
| Kong 2020 | A comprehensive evaluation of association between homocysteine levels and single nucleotide polymorphisms with hypertension risk: A protocol of systematic review and network meta-analysis | 2020 | No data about hyperhomocysteinemia |
| Kong 2022 | Association of MTHFR Polymorphisms with H-Type Hypertension: A Systemic Review and Network Meta-Analysis of Diagnostic Test Accuracy | 2022 | No data about hyperhomocysteinemia |
| Kouroglou 2019 | Vitamin B12 insufficiency is associated with increased risk of gestational diabetes mellitus: a systematic review and meta-analysis | 2019 | No data about hyperhomocysteinemia |
| Kouroglou 2019 | Vitamin B12 deficiency is associated with higher risk of gestational diabetes: a systematic review and meta-analysis | 2019 | No data about hyperhomocysteinemia |
| Kumar 2018 | MTHFR C677T polymorphism and risk of esophageal cancer: An updated meta-analysis | 2018 | No data about hyperhomocysteinemia |
| Kumar 2022 | The Role of Circulating Protein and Metabolite Biomarkers in the Development of Pancreatic Ductal Adenocarcinoma (PDAC): A Systematic Review and Meta-analysis | 2022 | No data about hyperhomocysteinemia |
| Kuźma 2018 | Which Risk Factors Causally Influence Dementia? A Systematic Review of Mendelian Randomization Studies | 2018 | No data about hyperhomocysteinemia |
| Lai 2017 | Peripheral inflammatory markers in Alzheimer's disease: A systematic review and meta-analysis of 175 studies | 2017 | No data about hyperhomocysteinemia |
| Lauretta 2022 | Homocysteine Plasmatic Concentration in Brain-Injured Neurocritical Care Patients: Systematic Review of Clinical Evidence | 2022 | Doubleds |
| Lauw 2013 | Cerebral venous thrombosis and thrombophilia: A systematic review and meta-analysis | 2013 | No data about hyperhomocysteinemia |
| Lee 2010 | Efficacy of homocysteine-lowering therapy with folic Acid in stroke prevention: a meta-analysis | 2010 | No data about hyperhomocysteinemia |
| Lee 2019 | Association between homocysteine levels and psoriasis: A metaanalysis | 2019 | doubled |
| Lei 2018 | Association between homocysteine level and the risk of diabetic retinopathy: A systematic review and meta-analysis | 2018 | doubled |
| Lewis 2005 | Meta-analysis of MTHFR 677C->T polymorphism and coronary heart disease: does totality of evidence support causal role for homocysteine and preventive potential of folate? | 2005 | No data about hyperhomocysteinemia |
| Li 2014 | Effects of <i>MTHFR</i> Genetic Polymorphisms on Toxicity and Clinical Response of Irinotecan-Based Chemotherapy in Patients with Colorectal Cancer | 2014 | No data about hyperhomocysteinemia |
| Li 2015 | Association between MTHFR C677T polymorphism and congenital heart disease A family-based meta-analysis | 2015 | No data about hyperhomocysteinemia |
| Li 2015 | Meta-analysis of clinical trials of folic acid, Vitamin B12 and B6 supplementation on plasma homocysteine level and risk of cardiovascular disease | 2015 | Wrong study design |
| Li 2016 | Plasma Homocysteine, Serum Folic Acid, Serum Vitamin B<sub>12</sub>, Serum Vitamin B<sub>6</sub>, MTHFR, and Risk of Normal-Tension Glaucoma | 2016 | doubled |
| Li 2016 | Does genistein lower plasma lipids and homocysteine levels in postmenopausal women? A meta-analysis | 2016 | No data about hyperhomocysteinemia |
| Li 2016 | Meta-analysis of association between MTHFR C677T polymorphism and risk of myocardial infarction: evidence from forty-four case-control studies | 2016 | No data about hyperhomocysteinemia |
| Li 2017 | Association between Serum Homocysteine Level and Obstructive Sleep Apnea: A Meta-Analysis | 2017 | doubled |
| Li 2019 | Plasma total homocysteine and risk of hypertension in pregnancy: A mendelian randomization study | 2019 | No data about hyperhomocysteinemia |
| Li 2020 | Meta-analysis of retinal vein occlusion risk factors | 2020 | No data about hyperhomocysteinemia |
| Li 2021 | The effect of metformin on homocysteine levels in patients with polycystic ovary syndrome: A systematic review and meta-analysis | 2021 | No data about hyperhomocysteinemia |
| Li 2023 | Modifiable vascular risk factors for outcomes in patients with CADASIL: A literature review and meta-analysis of cross-sectional analyzes | 2023 | No data about hyperhomocysteinemia |
| Li 2024 | Serum Homocysteine and Atrial Fibrillation Recurrence after Catheter Ablation: A Meta-Analysis of 11 Cohort Studies Involving 2147 Patients | 2024 | full-text not available |
| Li 2024 | Serum vitamins and homocysteine levels in autoimmune liver disease: A systematic review and meta-analysis | 2024 | No data about hyperhomocysteinemia |
| Li 2024 | Zhishi Xiebai Guizhi Decoction for coronary heart disease: A systematic review and meta-analysis | 2024 | Wrong study design |
| Liampas 2020 | Pyridoxine, folate and cobalamin for migraine: A systematic review | 2020 | Wrong study design |
| Liang 2022 | Homocysteine level at the acute stage of ischemic stroke as a biomarker of poststroke depression: A systematic review and meta-analysis | 2022 | doubled |
| Liang 2023 | Homocysteine level at the acute stage of ischemic stroke as a biomarker of poststroke depression: A systematic review and meta-analysis | 2023 | doubled |
| Liao 2025 | Potential biomarkers for cerebral small vessel disease with cognitive impairment: a systematic review and meta-analysis | 2025 | doubled |
| Lim 2024 | Patent foramen ovale closure in ischemic stroke patients with and without thrombophilia: a systematic review and meta-analyses | 2024 | wrong study design |
| Lin 2024 | Causal Relationships between Homocysteine and the Polycystic Ovary Syndrome: A Mendelian Randomization Analysis | 2024 | No data about hyperhomocysteinemia |
| Liu 2014 | The effect of homocysteine-lowering therapy with folic acid on flow-mediated vasodilation in patients with coronary artery disease: Ameta-analysis of randomized controlled trials | 2014 | doubled |
| Liu 2014 | <i>MTHFR</i> C677T polymorphism and migraine risk: A meta-analysis | 2014 | Wrong study design |
| Liu 2021 | 5,10-methylenetetrahydrofolate reductase C677T gene polymorphism and peripheral arterial disease: A meta-analysis | 2021 | No data about hyperhomocysteinemia |
| Liu 2021 | A Mendelian Randomization Study of Plasma Homocysteine Levels and Cerebrovascular and Neurodegenerative Diseases | 2021 | No data about hyperhomocysteinemia |
| Liu 2022 | Association between MTR A2756G polymorphism and susceptibility to congenital heart disease: A meta-analysis | 2022 | Wrong study design |
| Luo 2018 | Associations of the <i>MTHFR</i> rs1801133 polymorphism with coronary artery disease and lipid levels: a systematic review and updated meta-analysis | 2018 | No data about hyperhomocysteinemia |
| Ma 2024 | Blood biomarkers for post-stroke cognitive impairment: A systematic review and meta-analysis | 2024 | doubled |
| Ma 2024 | Prevalence and Risk Factors of Poststroke Cognitive Impairment: A Systematic Review and Meta-Analysis | 2024 | No data about hyperhomocysteinemia |
| Mahmood 2015 | Effects of folic acid supplementation on homocysteine level as primary prevention of heart disease: a systematic review | 2015 | No data about hyperhomocysteinemia |
| Malouf 2008 | Folic acid with or without vitamin B12 for the prevention and treatment of healthy elderly and demented people | 2008 | No data about hyperhomocysteinemia |
| Mandaviya 2017 | Genetically defined elevated homocysteine levels do not result in widespread changes of DNA methylation in leukocytes | 2017 | No data about hyperhomocysteinemia |
| Mangoni 2024 | Transsulfuration and folate pathways in rheumatoid arthritis: A systematic review and meta-analysis | 2024 | No data about hyperhomocysteinemia |
| Mann 2013 | Vascular thrombosis and vascular thrombo-embolism in inflammatory bowel disease: Systematic evaluation of 52,855 cases with meta-analysis | 2013 | No data about hyperhomocysteinemia |
| Mantovani 2024 | Biomarkers for cognitive impairment in alpha-synucleinopathies: an overview of systematic reviews and meta-analyses | 2024 | No data about hyperhomocysteinemia |
| Mao 2015 | One-carbon metabolic factors and risk of renal cell cancer: A meta-analysis | 2015 | No data about hyperhomocysteinemia |
| Martí-Carvajal 2013 | Homocysteine-lowering interventions for preventing cardiovascular events | 2013 | No data about hyperhomocysteinemia |
| Martí-Carvajal 2015 | Homocysteine-lowering interventions for preventing cardiovascular events | 2015 | No data about hyperhomocysteinemia |
| Martí-Carvajal 2017 | Homocysteine-lowering interventions for preventing cardiovascular events | 2017 | No data about hyperhomocysteinemia |
| Martin 2019 | A systematic review and meta-analysis of molecular biomarkers associated with early neurological deterioration following acute stroke | 2019 | No data about hyperhomocysteinemia |
| Martínez-Ceballos 2021 | Coronary calcium in autoimmune diseases: A systematic literature review and meta-analysis | 2021 | No data about hyperhomocysteinemia |
| Marwaha 2025 | Unlocking the Vitamin Puzzle: Investigating Levels in People With Alzheimer's Disease Versus Healthy Controls Through Systematic Review and Network Meta-Analysis | 2025 | No data about hyperhomocysteinemia |
| Matsuo 2024 | Association between Psoriasis and MTHFR polymorphisms: a systematic review and meta-analysis | 2024 | No data about hyperhomocysteinemia |
| McColgan 2008 | The genetics of carotid dissection: meta-analysis of a MTHFR/C677T common molecular variant | 2008 | No data about hyperhomocysteinemia |
| McColl 2013 | A systematic review of the nutritional status of women of a childbearing age with severe mental illness | 2013 | No data about hyperhomocysteinemia |
| McGimpsey 2009 | Homocysteine, Methylenetetrahydrofolate Reductase C677T Polymorphism, and Risk of Retinal Vein Occlusion: A Meta-analysis | 2009 | doubled |
| McNulty 2008 | Homocysteine, B-vitamins and CVD | 2008 | No data about hyperhomocysteinemia |
| Mehlig 2013 | The association between plasma homocysteine and coronary heart disease is modified by the MTHFR 677C>T polymorphism | 2013 | No data about hyperhomocysteinemia |
| Mendoza-Pinto 2020 | The Impact of Antimalarial Agents on Traditional and Non-traditional Cardiovascular Markers in Patients with Systemic Lupus Erythematosus: A Systematic Review and Meta-analysis | 2020 | No data about hyperhomocysteinemia |
| Meng 2016 | Association between high serum homocysteine levels and biochemical characteristics in women with polycystic ovarian syndrome: A systematic review and meta-analysis | 2016 | doubled |
| Merashli 2022 | Plasma Homocysteine in Behcet's Disease: A Systematic Review and Meta-Analysis | 2022 | full-text not available |
| MichelleWiest 2019 | An effect-size surface approach to meta-analysis | 2019 | No data about hyperhomocysteinemia |
| Mignini 2005 | Mapping the theories of preeclampsia: the role of homocysteine | 2005 | Wrong study design |
| Mohammad 2016 | Clinical utility of folate pathway genetic polymorphisms in the diagnosis of autism spectrum disorders | 2016 | No data about hyperhomocysteinemia |
| Møller 2000 | A meta-analysis of cerebrovascular disease and hyperhomocysteinaemia | 2000 | full-text not available |
| Nantachai 2021 | Oxidative stress and antioxidant defenses in mild cognitive impairment: A systematic review and meta-analysis | 2021 | doubled |
| Nantachai 2022 | Oxidative stress and antioxidant defenses in mild cognitive impairment: A systematic review and meta-analysis | 2022 | doubled |
| Nash 2019 | Interaction between plasma homocysteine and the <i>MTHFR c.677C</i> &gt; <i>T</i> polymorphism is associated with site-specific changes in DNA methylation in humans | 2019 | No data about hyperhomocysteinemia |
| Neves 2023 | Genetic polymorphisms of the folate pathway in amyotrophic lateral sclerosis and multiple sclerosis: a systematic review and meta-analysis | 2023 | No data about hyperhomocysteinemia |
| Nguyen 2023 | Sex Differences in the Level of Homocysteine in Alzheimer’s Disease and Parkinson’s Disease Patients: A Meta-Analysis | 2023 | No data about hyperhomocysteinemia |
| Ni 2014 | Meta - analysis of plasma homocysteine levels in valproic acid treated patients with epilepsy | 2014 | No data about hyperhomocysteinemia |
| Nie 2014 | Hyperhomocysteinemia and risk of cognitive decline: A meta-analysis of prospective cohort studies | 2014 | doubled |
| Niklewicz 2024 | A systematic review and meta-analysis of functional vitamin B12 status among adult vegans | 2024 | No data about hyperhomocysteinemia |
| Nishi 2014 | Meta-analyses of blood homocysteine levels for gender and genetic association studies of the MTHFR C677T polymorphism in schizophrenia | 2014 | No data about hyperhomocysteinemia |
| Nishi 2014 | Meta-analyses of Blood Homocysteine Levels for Gender and Genetic Association Studies of the <i>MTHFR</i> C677T Polymorphism in Schizophrenia | 2014 | No data about hyperhomocysteinemia |
| Obersby 2012 | Homocysteine and vitamin B12 status of vegetarians as compared to omnivores: A potential health hazard? | 2012 | Wrong study design |
| Obersby 2012 | Are vegetarians susceptible to primary cardiovascular disease, due to a lack or absence of dietary animal produce? | 2012 | Wrong study design |
| Obersby 2013 | Plasma total homocysteine status of vegetarians compared with omnivores: A systematic review and meta-Analysis | 2013 | No data about hyperhomocysteinemia |
| Ozkose 2025 | Factors Associated with Thrombosis in Behçet Syndrome: A Systematic Review and Meta-Analysis | 2025 | No data about hyperhomocysteinemia |
| Pan 2019 | Homocysteine, vitamin B12, and folate levels in patients with multiple sclerosis in Chinese population: A case-control study and meta-analysis | 2019 | doubled |
| Papadaki 2020 | The effect of the mediterranean diet on metabolic health: A systematic review and meta-analysis of controlled trials in adults | 2020 | No data about hyperhomocysteinemia |
| Park 2016 | Effect of B-vitamins on stroke risk among individuals with vascular disease who are not on antiplatelets: A meta-analysis | 2016 | No data about hyperhomocysteinemia |
| Park 2021 | Causal effects of homocysteine, folate, and cobalamin on kidney function: A mendelian randomization study | 2021 | wrong study design |
| Parthasarathy 2023 | The role of prognostic biomarkers and their implications in early detection of preeclampsia: A systematic review | 2023 | No data about hyperhomocysteinemia |
| Pecze 2020 | Meta-analysis of metabolites involved in bioenergetic pathways reveals a pseudohypoxic state in Down syndrome | 2020 | No data about hyperhomocysteinemia |
| Peerbooms 2011 | Meta-analysis of MTHFR gene variants in schizophrenia, bipolar disorder and unipolar depressive disorder: Evidence for a common genetic vulnerability? | 2011 | No data about hyperhomocysteinemia |
| PinheiroVolp 2015 | Hepatic inflammatory biomarkers and its link with obesity and chronic diseases | 2015 | No data about hyperhomocysteinemia |
| Pinna 2015 | Homocysteine and risk of wet age-related macular degeneration: A meta-analysis | 2015 | doubled |
| Pinzon 2023 | The role of homocysteine levels as a risk factor of ischemic stroke events: a systematic review and meta-analysis | 2023 | doubled |
| Pooja 2015 | MTHFR 677C&gt;T Polymorphism and the Risk of Breast Cancer: Evidence from an Original Study and Pooled Data for 28031 Cases and 31880 Controls | 2015 | Wrong study design |
| Poorzand 2019 | Risk factors of premature coronary artery disease in Iran: A systematic review and meta-analysis | 2019 | Wrong study design |
| Potter 2008 | The effect of long-term homocysteine-lowering on carotid intima-media thickness and flow-mediated vasodilation in stroke patients: a randomized controlled trial and meta-analysis | 2008 | Wrong study design |
| Prado 2023 | The Effects of Methylfolate on Cognitive Decline and Dementia: A Protocol for Systematic Review and Meta-Analysis | 2023 | No data about hyperhomocysteinemia |
| Qi 2014 | Methylenetetrahydrofolate reductase C677T gene mutation and hyperhomocysteinemia in Budd-Chiari syndrome and portal vein thrombosis: A systematic review and meta-analysis of observational studies | 2014 | No data about hyperhomocysteinemia |
| Qi 2015 | Association between <i>MTHFR</i> 677C/T polymorphism and psoriasis risk: a meta-analysis | 2015 | No data about hyperhomocysteinemia |
| Qin 2013 | Homocysteine-lowering therapy with folic acid is effective in cardiovascular disease prevention in patients with kidney disease: A meta-analysis of randomized controlled trials | 2013 | No data about hyperhomocysteinemia |
| Quan 2023 | Association between the risk and severity of Parkinson’s disease and plasma homocysteine, vitamin B12 and folate levels: a systematic review and meta-analysis | 2023 | doubled |
| Rademacher 2023 | Molecular biomarkers and cognitive impairment in multiple sclerosis: State of the field, limitations, and future direction – A systematic review and meta-analysis | 2023 | doubled |
| Rafnsson 2011 | Is a low blood level of vitamin B12 a cardiovascular and diabetes risk factor? A systematic review of cohort studies | 2011 | No data about hyperhomocysteinemia |
| Rahnemayan 2023 | An umbrella review of systematic reviews with meta-analysis on the role of vitamins in Parkinson’s disease | 2023 | No data about hyperhomocysteinemia |
| Rai 2016 | Association of methylenetetrahydrofolate reductase (<i>MTHFR</i>) gene C677T polymorphism with autism: evidence of genetic susceptibility | 2016 | wrong study design |
| Rai 2017 | Methylenetetrahydrofolate Reductase (MTHFR) C677T Polymorphism and Alzheimer Disease Risk: a Meta-Analysis | 2017 | No data about hyperhomocysteinemia |
| Raina 2022 | “Association of MTHFR and MS/MTR gene polymorphisms with congenital heart defects in North Indian population (Jammu and Kashmir): a case–control study encompassing meta-analysis and trial sequential analysis” | 2022 | No data about hyperhomocysteinemia |
| Ranasinghe 2025 | Hypertension in Sri Lanka: A systematic review of prevalence, control, care delivery and challenges | 2025 | No data about hyperhomocysteinemia |
| Ray 2002 | Common C677T polymorphism of the methylenetetrahydrofolate reductase gene and the risk of venous thromboembolism: meta-analysis of 31 studies | 2002 | No data about hyperhomocysteinemia |
| Ren 2017 | MTHFR C677T, A1298C and MS A2756G Gene Polymorphisms and Male Infertility Risk in a Chinese Population: A Meta-Analysis | 2017 | No data about hyperhomocysteinemia |
| Rogne 2017 | Associations of Maternal Vitamin B12 Concentration in Pregnancy With the Risks of Preterm Birth and Low Birth Weight: A Systematic Review and Meta-Analysis of Individual Participant Data | 2017 | No data about hyperhomocysteinemia |
| Roostaei 2016 | Genetic influence of plasma homocysteine level on alzheimer's disease | 2016 | Wrong study design |
| Rossignol 2021 | The effectiveness of cobalamin (B12) treatment for autism spectrum disorder: A systematic review and meta-analysis | 2021 | No data about hyperhomocysteinemia |
| Roufael 2023 | Folate-Methionine Cycle Disruptions in ASD Patients and Possible Interventions: A Systematic Review | 2023 | No data about hyperhomocysteinemia |
| Ruan 2015 | Effect of B vitamin (Folate, B6, and B12) supplementation on osteoporotic fracture and bone turnover markers: A meta-analysis | 2015 | No data about hyperhomocysteinemia |
| Rubinstein 2005 | A systematic review of the risk factors for cervical artery dissection | 2005 | No data about hyperhomocysteinemia |
| Salagre 2017 | Homocysteine as a peripheral biomarker in bipolar disorder: A meta-analysis | 2017 | full-text not available |
| Salo 2019 | Confining the Concept of Vascular Depression to Late-Onset Depression: A Meta-Analysis of MRI-Defined Hyperintensity Burden in Major Depressive Disorder and Bipolar Disorder | 2019 | No data about hyperhomocysteinemia |
| Sanguankeo 2016 | Association between homocysteine and white coat hypertension: A systematic review and meta-analysis | 2016 | conference abstract |
| Sansone 2018 | Serum homocysteine levels in men with and without erectile dysfunction: A systematic review and meta-analysis | 2018 | Wrong study design |
| Sansone 2018 | Serum homocysteine levels in men with and without erectile dysfunction: A systematic review and meta-analysis | 2018 | Wrong study design |
| Sansone 2019 | Serum Homocysteine Levels in Men with and without Erectile Dysfunction: A Systematic Review and Meta-analysis | 2019 | doubled |
| Sarecka-Hujar 2012 | The TT genotype of methylenetetrahydrofolate reductase 677C>T polymorphism increases the susceptibility to pediatric ischemic stroke: Meta-analysis of the 822 cases and 1,552 controls | 2012 | No data about hyperhomocysteinemia |
| Sarecka-Hujar 2012 | The TT genotype of methylenetetrahydrofolate reductase 677C&gt;T polymorphism increases the susceptibility to pediatric ischemic stroke: meta-analysis of the 822 cases and 1,552 controls | 2012 | No data about hyperhomocysteinemia |
| Setién-Suero 2016 | Homocysteine and cognition: A systematic review of 111 studies | 2016 | Doubleds |
| Shen 2015 | Associations between B vitamins and Parkinson’s disease | 2015 | No data about hyperhomocysteinemia |
| Shen 2015 | Associations between homocysteine, folic acid, vitamin B12 and Alzheimer's disease: Insights from meta-analyses | 2015 | No data about hyperhomocysteinemia |
| Shiao 2016 | Meta-Prediction of <i>MTHFR</i> Gene Polymorphism Mutations and Associated Risk for Colorectal Cancer | 2016 | No data about hyperhomocysteinemia |
| Shiao 2018 | Meta-analysis of homocysteine-related factors on the risk of colorectal cancer | 2018 | Doubleds |
| Shu 2015 | Association between the methylenetetrahydrofolate reductase gene C677T polymorphism and sudden sensorineural hearing loss: a meta-analysis | 2015 | No data about hyperhomocysteinemia |
| Silva 2019 | Effects of Creatine Supplementation on Renal Function: A Systematic Review and Meta-Analysis | 2019 | No data about hyperhomocysteinemia |
| Singh 2012 | Folate Gene Polymorphisms <i>MTR</i> A2756G, <i>MTRR</i> A66G, and <i>BHMT</i> G742A and Risk for Coronary Artery Disease: A Meta-Analysis | 2012 | No data about hyperhomocysteinemia |
| Skrobot 2016 | A Validation Study of Vascular Cognitive Impairment Genetics Meta-Analysis Findings in an Independent Collaborative Cohort | 2016 | No data about hyperhomocysteinemia |
| Sohouli 2024 | A comprehensive review and meta-regression analysis of randomized controlled trials examining the impact of vitamin B<sub>12</sub> supplementation on homocysteine levels | 2024 | Wrong study design |
| Song 2021 | Association Between Vitamin D Status and Undernutrition Indices in Children: A Systematic Review and Meta-Analysis of Observational Studies | 2021 | No data about hyperhomocysteinemia |
| Sun 2021 | No causal association between plasma homocysteine levels and atrial fibrillation: A Mendelian randomization study | 2021 | Wrong study design |
| Tabassum 2012 | Genetic variant of amd1 is associated with obesity in urban indian children | 2012 | No data about hyperhomocysteinemia |
| Takagi 2015 | A meta-analysis of circulating homocysteine levels in subjects with versus without abdominal aortic aneurysm | 2015 | full-text not available |
| Takagi 2015 | A meta-analysis of circulating homocysteine levels in subjects with <i>versus</i> without abdominal aortic aneurysm | 2015 | No data about hyperhomocysteinemia |
| Tan 2013 | Venous and arterial disease in inflammatory bowel disease | 2013 | No data about hyperhomocysteinemia |
| Tan 2023 | Correlation between Vitamin B12 and Mental Health in Children and Adolescents: A Systematic Review and Meta-analysis | 2023 | No data about hyperhomocysteinemia |
| Tang 2022 | Association of kidney function and brain health: A systematic review and meta-analysis of cohort studies | 2022 | Wrong study design |
| Tian 2017 | Folic Acid Supplementation for Stroke Prevention in Patients With Cardiovascular Disease | 2017 | Wrong study design |
| Tian 2021 | Associations of hematological and biochemical markers with intracranial atherosclerosis: A systematic review and meta-analysis of observational studies | 2021 | conference abstract |
| Tsai 2019 | Serum homocysteine, folate, and vitamin B<sub>12</sub> levels in patients with vitiligo and their potential roles as disease activity biomarkers: A systematic review and meta-analysis | 2019 | doubled |
| Tsai 2019 | Serum homocysteine, folate and vitamin B<sub>12</sub> levels in patients with psoriasis: a systematic review and meta-analysis | 2019 | No data about hyperhomocysteinemia |
| Tsai 2021 | Serum Homocysteine, Folate, and Vitamin B<sub>12</sub> Levels in Patients with Systemic Lupus Erythematosus: A Meta-Analysis and Meta-Regression | 2021 | full-text not available |
| Tuenter 2019 | Folate, vitamin B12, and homocysteine in smoking-exposed pregnant women: A systematic review | 2019 | No data about hyperhomocysteinemia |
| Ungprasert 2014 | Psoriasis and risk of venous thromboembolism: a systematic review and meta-analysis | 2014 | No data about hyperhomocysteinemia |
| Vahed 2024 | The Relationship Between Serum Folate and Homocysteine with Head and Neck Cancer: A Systematic Review and Meta-analysis | 2024 | No data about hyperhomocysteinemia |
| Valero 2007 | MTHFR C677T polymorphism and osteoporotic fractures | 2007 | No data about hyperhomocysteinemia |
| VanDam 2009 | Hyperhomocysteinemia and Alzheimer's disease: A systematic review | 2009 | doubled |
| vanKarnebeek 2012 | Treatable inborn errors of metabolism causing intellectual disability: a systematic literature review | 2012 | No data about hyperhomocysteinemia |
| vanWijngaarden 2013 | Vitamin B12, folate, homocysteine, and bone health in adults and elderly people: a systematic review with meta-analyses | 2013 | doubled |
| Verldeij-Hagoort 2007 | Hyperhomocysteinemia and <i>MTHFR</i> polymorphisms in association with orofacial clefts and congenital heart defects:: A meta-analysis | 2007 | doubled |
| Vidale 2017 | The association between homocysteine and carotid restenosis after endarterectomy: A meta-analysis | 2017 | conference abstract |
| Virk 2023 | The Effectiveness of Polypill for the Prevention of Cardiovascular Disease: A Meta-Analysis of Randomized Controlled Trials | 2023 | No data about hyperhomocysteinemia |
| Wald 1998 | Homocysteine and ischemic heart disease: results of a prospective study with implications regarding prevention | 1998 | Wrong study design |
| Wald 2002 | Homocysteine and cardiovascular disease: evidence on causality from a meta-analysis | 2002 | Wrong study design |
| Wald 2011 | Reconciling the evidence on serum homocysteine and ischaemic heart disease: a meta-analysis | 2011 | No data about hyperhomocysteinemia |
| Wang 2012 | Meta-analysis of the relationship between hyperhomocysteinemia and multiple sclerosis | 2012 | conference abstract |
| Wang 2014 | Meta-analysis of plasma homocysteine content and cognitive function in elderly patients with alzheimer’s disease and vascular dementia | 2014 | doubled |
| Wang 2017 | Serum folate, vitamin B12 levels and diabetic peripheral neuropathy in type 2 diabetes: A meta-analysis | 2017 | No data about hyperhomocysteinemia |
| Wang 2017 | Homocysteine: A potential common route for cardiovascular risk and DNA methylation in psoriasis | 2017 | Wrong study design |
| Wang 2019 | Association between homocysteine and nonalcoholic fatty liver disease: A systematic review with trial sequential analysis | 2019 | conference abstract |
| Wang 2021 | Association Between Homocysteine and Type 2 Diabetes Mellitus: a Systematic Review and Meta-analysis | 2021 | doubled |
| Wang 2021 | Causal effects of homocysteine levels on the changes of bone mineral density and risk for bone fracture: A two-sample mendelian randomization study | 2021 | Wrong study design |
| Wang 2022 | Systematic review and meta-analysis of the correlation between plasma homocysteine levels and coronary heart disease | 2022 | doubled |
| Wang 2022 | Relationship between serum homocysteine, fibrinogen, lipoprotein-a level, and peripheral arterial disease: a dose–response meta-analysis | 2022 | No data about hyperhomocysteinemia |
| Wang 2022 | Efficacy and Safety of Electroacupuncture in the Treatment of Cerebral Infarction: Systematic Review and Meta-Analysis | 2022 | No data about hyperhomocysteinemia |
| Wang 2022 | Higher levels of peripheral blood glucose in the acute stage of stroke increase the risk of post-stroke depression: A systematic review and meta-analysis | 2022 | No data about hyperhomocysteinemia |
| Wang 2022 | The Beneficial Effect of a Healthy Dietary Pattern on Androgen Deprivation Therapy-Related Metabolic Abnormalities in Patients with Prostate Cancer: A Meta-Analysis Based on Randomized Controlled Trials and Systematic Review | 2022 | Wrong study design |
| Wang 2023 | Identification of methylation-regulated genes modulating microglial phagocytosis in hyperhomocysteinemia-exacerbated Alzheimer’s disease | 2023 | Wrong study design |
| Wang 2024 | Effectiveness of DL-3-n-butylphthalide in the treatment of poststroke cognitive impairment and its associated predictive cytokines: a systematic review and meta-analysis | 2024 | Wrong study design |
| Wartena 2023 | Polycystic ovary syndrome and recurrent pregnancy loss, a review of literature | 2023 | Wrong study design |
| Weiner 2014 | Polymorphisms in folate-metabolizing genes and risk of idiopathic male infertility: a study on a Russian population and a meta-analysis | 2014 | Wrong study design |
| Wirthensohn 2023 | Biochemical, Nutritional, and Clinical Parameters of Vitamin B12 Deficiency in Infants: A Systematic Review and Analysis of 292 Cases Published between 1962 and 2022 | 2023 | Wrong study design |
| Wiseman 2014 | Blood markers of coagulation, fibrinolysis, endothelial dysfunction and inflammation in lacunar stroke versus non-lacunar stroke and non-stroke: Systematic review and meta-analysis | 2014 | Wrong study design |
| Wolters 2020 | Effects of phytoestrogen supplementation on intermediate cardiovascular disease risk factors among postmenopausal women: A meta-analysis of randomized controlled trials | 2020 | No data about hyperhomocysteinemia |
| Wu 2013 | Association between MTHFR C677T polymorphism and depression: An updated meta-analysis of 26 studies | 2013 | No data about hyperhomocysteinemia |
| Wu 2014 | Association between methylenetetrahydrofolate reductase C677T polymorphism and epilepsy susceptibility: A meta-analysis | 2014 | No data about hyperhomocysteinemia |
| Wu 2017 | Plasma homocysteine levels and risk of vascular dementia: A Mendelian randomization study | 2017 | Wrong study design |
| Wu 2020 | Association of homocysteine level with risk of stroke: A dose–response meta-analysis of prospective cohort studies | 2020 | doubled |
| Wu 2023 | Association Between Maternal Factors and Risk of Congenital Heart Disease in Offspring: A Systematic Review and Meta-Analysis | 2023 | doubled |
| Xia 2012 | Meta-analysis of the methylenetetrahydrofolate reductase C677T polymorphism and susceptibility to pre-eclampsia | 2012 | No data about hyperhomocysteinemia |
| Xie 2017 | Association of plasma homocysteine, vitamin B12 and folate levels with cognitive function in Parkinson's disease: A meta-analysis | 2017 | doubled |
| Xin-Shuai 2024 | Meta-Analysis of the efficacy and safety of Ginkgolide Meglumine Injection combined with Butylphthalide in the treatment of Acute Ischemic Stroke | 2024 | Wrong study design |
| Xu 2012 | Homocysteine, B vitamins, methylenetetrahydrofolate reductase gene, and risk of primary open-angle glaucoma: A meta-analysis | 2012 | doubled |
| Xu 2016 | Evaluation of an Association of Blood Homocysteine Levels with Gastric Cancer Risk from 27 Case-Control Studies | 2016 | No data about hyperhomocysteinemia |
| Xu 2016 | Association between <i>BHMT</i> gene rs3733890 polymorphism and cancer risk: evidence from a meta-analysis | 2016 | No data about hyperhomocysteinemia |
| Xu 2016 | Effect of omega-3 fatty acid supplementation on serum lipids and vascular inflammation in patients with end-stage renal disease: a meta-analysis | 2016 | Wrong study design |
| Xu 2017 | Association between Methionine Synthase Reductase A66G Polymorphism and Male Infertility: A Meta-Analysis | 2017 | No data about hyperhomocysteinemia |
| Xu 2018 | Lack of association between <i>MTHFR</i> <i>C677T</i> Gene polymorphism with alcohol dependence: A meta-analysis of case-control studies | 2018 | No data about hyperhomocysteinemia |
| Xu 2019 | Plasma homocysteine levels may be associated with the subtypes of ischemic stroke: A meta-analysis | 2019 | doubled |
| Xu 2021 | Serum folic acid levels and erectile dysfunction: A meta-analysis and systematic review | 2021 | No data about hyperhomocysteinemia |
| Xu 2024 | Effects of folic acid supplementation on cognitive impairment: A meta-analysis of randomized controlled trials | 2024 | Wrong study design |
| Xue 2018 | Association of homocysteine and contrast-induced nephropathy: A meta-analysis | 2018 | No data about hyperhomocysteinemia |
| Yadav 2013 | Detailed Analysis of Gene Polymorphisms Associated with Ischemic Stroke in South Asians | 2013 | No data about hyperhomocysteinemia |
| Yadav 2016 | Role of MTHFR C677T gene polymorphism in the susceptibility of schizophrenia: An updated meta-analysis | 2016 | No data about hyperhomocysteinemia |
| Yadav 2016 | Role of <i>MTHFR</i> C677T gene polymorphism in the susceptibility of schizophrenia: An updated meta-analysis | 2016 | No data about hyperhomocysteinemia |
| Yadav 2021 | Distribution of Methionine Synthase Reductase (MTRR) Gene A66G Polymorphism in Indian Population | 2021 | No data about hyperhomocysteinemia |
| Yajnik 2014 | Maternal homocysteine in pregnancy and offspring birthweight: Epidemiological associations and Mendelian randomization analysis | 2014 | Wrong study design |
| Yang 2015 | Prevalence of hyperhomocysteinemia in china: A systematic review and meta-analysis | 2015 | No data about hyperhomocysteinemia |
| Yang 2015 | Association between <i>MTHFR</i> C677T polymorphism and neural tube defect risks: A comprehensive evaluation in three groups of NTD patients, mothers, and fathers | 2015 | Wrong study design |
| Yang 2021 | Circulating trimethylamine N-oxide in association with diet and cardiometabolic biomarkers: An international pooled analysis | 2021 | No data about hyperhomocysteinemia |
| Yang 2022 | Association of methylenetetrahydrofolate reductase (MTHFR) gene polymorphisms (C677T and A1298C) with thyroid dysfunction: A meta-analysis and trial sequential analysis | 2022 | No data about hyperhomocysteinemia |
| Yang 2025 | Prediction Models for Post-Stroke Cognitive Impairment: A Systematic Review and Meta-Analysis | 2025 | No data about hyperhomocysteinemia |
| Ye 2021 | N-acetylcysteine for chronic kidney disease: A systematic review and meta-analysis | 2021 | Wrong study design |
| Yi 2013 | Efficacy of folic acid supplementation alone on endothelial function and plasma homocysteine concentration in coronary artery disease: A meta-analysis of randomized controlled trials | 2013 | Wrong study design |
| Ying 2017 | Meta-analysis of the relationship between plasma homocysteine levels and carbamazepine monotherapy in patients with epilepsy | 2017 | Wrong study design |
| Yoshimi 2010 | Gene-wide association study between the methylenetetrahydrofolate reductase gene (<i>MTHFR</i>) and schizophrenia in the Japanese population, with an updated meta-analysis on currently available data | 2010 | No data about hyperhomocysteinemia |
| Yu 2013 | Association between <i>Methylenetetrahydrofolate Reductase C677T</i> Polymorphism and Susceptibility to Cervical Cancer: A Meta-Analysis | 2013 | No data about hyperhomocysteinemia |
| Yu 2014 | Association Between Methionine Synthase Reductase A66G Polymorphism and the Risk of Congenital Heart Defects: Evidence From Eight Case–Control Studies | 2014 | No data about hyperhomocysteinemia |
| Yu 2020 | Evidence-based prevention of Alzheimer's disease: Systematic review and meta-analysis of 243 observational prospective studies and 153 randomised controlled trials | 2020 | No data about hyperhomocysteinemia |
| Yu 2022 | Causal effects of homocysteine levels on the components of sarcopenia: A two-sample mendelian randomization study | 2022 | Wrong study design |
| Yu 2023 | Homocysteine levels in patients with coronary slow flow phenomenon: A meta-analysis | 2023 | doubled |
| Yuan 2017 | Genetic polymorphism of methylenetetrahydrofolate reductase as a potential risk factor for congenital heart disease: A meta-analysis in Chinese pediatric population | 2017 | No data about hyperhomocysteinemia |
| Yue 2021 | Maternal intake of caffeinated products and birth defects: a systematic review and meta-analysis of observational studies | 2021 | No data about hyperhomocysteinemia |
| Zeng 2015 | The effect of folate fortification on folic acid-based homocysteine-lowering intervention and stroke risk: a meta-analysis | 2015 | Wrong study design |
| Zeng 2017 | Association between homocysteine levels and calcific aortic valve disease: A meta-analysis | 2017 | conference abstract |
| Zeng 2021 | Association between MTHFR polymorphisms (MTHFR C677T, MTHFR A1298C) and recurrent implantation failure: a systematic review and meta-analysis | 2021 | No data about hyperhomocysteinemia |
| Zesiewicz 2006 | The controversy concerning plasma homocysteine in Parkinson disease patients treated with levodopa alone or with entacapone: effects of vitamin status | 2006 | Wrong study design |
| Zhang 2013 | Effect of B-vitamin supplementation on stroke: A meta-analysis of randomized controlled trials | 2013 | Wrong study design |
| Zhang 2014 | Association of homocysteine, vitamin B12, and folate with bone mineral density in postmenopausal women: A meta-analysis | 2014 | doubled |
| Zhang 2014 | A meta-analysis of the relationship between MTHFR gene A1298C polymorphism and the risk of adult stroke | 2014 | No data about hyperhomocysteinemia |
| Zhang 2015 | Association Between MTHFR C677T Polymorphism and Venous Thromboembolism Risk in the Chinese Population: A Meta-Analysis of 24 Case-Controlled Studies | 2015 | No data about hyperhomocysteinemia |
| Zhang 2016 | Role of methylenetetrahydrofolate reductase 677C→T polymorphism in the development of myocardial infarction: evidence from an original study and updated meta-analysis | 2016 | No data about hyperhomocysteinemia |
| Zhang 2016 | Hyperhomocysteinemia results from and promotes hepatocellular carcinoma via CYP450 metabolism | 2016 | Wrong study design |
| Zhang 2016 | Metformin treatment and homocysteine: A systematic review and meta-analysis of randomized controlled trials | 2016 | Wrong study design |
| Zhang 2017 | Association between MTHFR A1298C polymorphism and male infertility: A meta-analysis | 2017 | No data about hyperhomocysteinemia |
| Zhang 2017 | Efficacy of Vitamin B Supplementation on Cognition in Elderly Patients with Cognitive-Related Diseases: A Systematic Review and Meta-Analysis | 2017 | Wrong study design |
| Zhang 2019 | Association between PON1 L55M polymorphisms and risk of coronary heart disease: a meta-analysis based on 46 case-control studies | 2019 | No data about hyperhomocysteinemia |
| Zhang 2019 | The association between maternal methylenetetrahydrofolate reductase C677T and A1298C polymorphism and birth defects and adverse pregnancy outcomes | 2019 | No data about hyperhomocysteinemia |
| Zhang 2020 | The association between homocysteine and ischemic stroke subtypes in Chinese: A meta-analysis | 2020 | doubled |
| Zhang 2020 | Vitamin B12, B6, or Folate and Cognitive Function in Community-Dwelling Older Adults: A Systematic Review and Meta-Analysis | 2020 | No data about hyperhomocysteinemia |
| Zhang 2021 | Association of homocysteine level with adverse outcomes in patients with acute ischemic stroke: A meta-analysis | 2021 | full-text not available |
| Zhang 2021 | Relationship between total plasma homocysteine and the risk of aneurysms - A meta-analysis | 2021 | full-text not available |
| Zhang 2021 | Associations of methylenetetrahydrofolate reductase gene (<i>MTHFR</i>) rs1801131 and rs1801133 polymorphisms with susceptibility to vitiligo: A meta-analysis | 2021 | No data about hyperhomocysteinemia |
| Zhang 2022 | Blood and CSF Homocysteine Levels in Alzheimer’s Disease: A Meta-Analysis and Meta-Regression of Case–Control Studies | 2022 | doubled |
| Zhang 2022 | Serum homocysteine level was elevated in ulcerative colitis and can be applied as diagnostic biomarker | 2022 | No data about hyperhomocysteinemia |
| Zhang 2024 | Folic acid supplementation for stroke prevention: A systematic review and meta-analysis of 21 randomized clinical trials worldwide | 2024 | Wrong study design |
| Zhao 2018 | The effects of folate supplementation on glucose metabolism and risk of type 2 diabetes: a systematic review and meta-analysis of randomized controlled trials | 2018 | Wrong study design |
| Zhao 2021 | Correlation of oxidative stress-related biomarkers with postmenopausal osteoporosis: a systematic review and meta-analysis | 2021 | No data about hyperhomocysteinemia |
| Zheng 2015 | Elevated serum homocysteine level in the development of diabetic peripheral neuropathy | 2015 | doubled |
| Zheng 2020 | Adjunctive folate for major mental disorders: A systematic review | 2020 | Wrong study design |
| Zheng 2023 | Effects of New Antiepileptic Drugs on Homocysteine in Epileptic Patients: A Systematic Review and Meta-Analysis | 2023 | Wrong study design |
| Zhong 2013 | Methylenetetrahydrofolate Reductase Gene Polymorphism and Risk of Type 2 Diabetes Mellitus | 2013 | No data about hyperhomocysteinemia |
| Zhou 2012 | Association between B-group vitamins and venous thrombosis: Systematic review and meta-analysis of epidemiological studies | 2012 | No data about hyperhomocysteinemia |
| Zhou 2020 | A meta-analysis of serum Hcy in diagnosis of diabetic nephropathy | 2020 | Doubleds |
| Zhou 2022 | Association of Vitamin B Status with Risk of Dementia in Cohort Studies: A Systematic Review and Meta-Analysis | 2022 | No data about hyperhomocysteinemia |
| Zhou 2023 | Mudan Granules for Diabetic Peripheral Neuropathy: A Systematic Review of Preclinical and Clinical Evidence | 2023 | Wrong study design |
| Zhu 2009 | [Meta-analysis on relationship between hyperhomocysteinemia and multiple sclerosis] | 2009 | language |
| Zhu 2011 | Meta-analysis of the relationship between homocysteine, vitamin B₁₂, folate, and multiple sclerosis | 2011 | doubled |
| Zhu 2011 | Meta-analysis of the relationship between homocysteine, vitamin B<sub>12</sub>, folate, and multiple sclerosis | 2011 | No data about hyperhomocysteinemia |
| Zhu 2013 | Association between MTHFR C677T, MTHFR A1298C and MS A2756G polymorphisms and risk of cervical intraepithelial neoplasia II/III and cervical cancer: a meta-analysis | 2013 | No data about hyperhomocysteinemia |
| Zhu 2015 | Association between the methylenetetrahydrofolate reductase (<i>MTHFR</i>) gene C677T polymorphism and ischemic stroke in the Chinese population: a meta-analysis | 2015 | No data about hyperhomocysteinemia |
| Zhu 2019 | Elevated homocysteine level and prognosis in patients with acute coronary syndrome: a meta-analysis | 2019 | full-text not available |
| Zhu 2019 | Association of methylenetetrahydrofolate reductase C677T and A1298C polymorphisms with genetic susceptibility to polycystic ovary syndrome: A PRISMA-compliant meta-analysis | 2019 | No data about hyperhomocysteinemia |
| Zhu 2024 | Association of Circulating Homocysteine Level with the Risk of Nephropathy in Type 2 Diabetes Mellitus: A Meta-Analysis | 2024 | full-text not available |
| Zhu 2025 | Association of Circulating Homocysteine Level with the Risk of Nephropathy in Type 2 Diabetes Mellitus: A Meta-Analysis | 2025 | full-text not available |
| Zinellu 2023 | A systematic review and meta-analysis of homocysteine concentrations in chronic obstructive pulmonary disease | 2023 | doubled |
| Zintzaras 2005 | The relationship between C677T methylenetetrahydrofolate reductase gene polymorphism and retinopathy in type 2 diabetes: a meta-analysis | 2005 | No data about hyperhomocysteinemia |
| Zintzaras 2006 | C677T and A1298C methylenetetrahydrofolate reductase gene polymorphisms in schizophrenia, bipolar disorder and depression: a meta-analysis of genetic association studies | 2006 | No data about hyperhomocysteinemia |
| Zintzaras 2007 | Maternal gene polymorphisms involved in folate metabolism and risk of Down syndrome offspring: a meta-analysis | 2007 | No data about hyperhomocysteinemia |
| Zis 2017 | Peripheral neuropathy in idiopathic Parkinson's disease: A systematic review | 2017 | No data about hyperhomocysteinemia |
| Zou 2022 | A systematic review and meta-analysis expounding the relationship between methylene tetrahydrofolate reductase gene polymorphism and the risk of intracerebral hemorrhage among populations | 2022 | No data about hyperhomocysteinemia |
| Zuin 2021 | Elevated Blood Homocysteine and Risk of Alzheimer’s Dementia: An Updated Systematic Review and Meta-Analysis Based on Prospective Studies | 2021 | doubled |

**Supplementary Table 4. List of included meta-analyses**

2002. Homocysteine and risk of ischemic heart disease and stroke: a meta-analysis. JAMA 288, 2015–2022.

Bala, R., Verma, R., Verma, P., Singh, V., Yadav, N., Rajender, S., Agrawal, N.R., Singh, K., 2021. Hyperhomocysteinemia and low vitamin B12 are associated with the risk of early pregnancy loss: A clinical study and meta-analyses. Nutrition Research 91, 57–66.

Bautista, L.E., Arenas, I.A., Peñuela, A., Martínez, L.X., 2002. Total plasma homocysteine level and risk of cardiovascular disease: a meta-analysis of prospective cohort studies. J Clin Epidemiol 55, 882–887.

Boushey, C.J., Beresford, S.A.A., Omenn, G.S., Motulsky, A.G., Medice, C., Univ Washington, D.E.S.W.A.U.S.A., 1995. A meta-analysis of plasma homocysteine as a risk factor for arteriosclerotic vascular disease and the potential preventive role of folic acid. International Conference on Homocysteine Metabolism, 245–249.

Cahill, M.T., Stinnett, S.S., Fekrat, S., 2003. Meta-analysis of plasma homocysteine, serum folate, serum vitamin B<sub>12</sub> and thermolabile MTHFR genotype as risk factors for retinal vascular occlusive disease. Investigative Ophthalmology & Visual Science 44, U683–U683.

Cao, H., Hu, X., Zhang, Q., Li, J., Wang, J., Shao, Y., Liu, B., Xin, S., 2014. Homocysteine level and risk of abdominal aortic aneurysm: A meta-analysis. PLoS ONE 9.

Carpenè, G., Negrini, D., Henry, B.M., Montagnana, M., Lippi, G., 2022. Homocysteine in coronavirus disease (COVID-19): a systematic literature review. Diagnosis (Berl) 9, 306–310.

Chen, W., Feng, J., Ji, P., Liu, Y., Wan, H., Zhang, J., 2023. Association of hyperhomocysteinemia and chronic kidney disease in the general population: a systematic review and meta-analysis. BMC Nephrology 24.

Chen, Y., Zou, H., Peng, M., Chen, Y., 2022. Association between homocysteine levels in acute stroke and poststroke depression: A systematic review and meta-analysis. Brain and Behavior 12.

Cheng, P., Li, H.M., Shen, Y.J., Wang, Y.W., Tang, X.L., 2024. Association between serum homocysteine and erectile dysfunction: a systematic review and meta-analysis. International Journal of Impotence Research.

Collin, S.M., Metcalfe, C., Refsum, H., Lewis, S.J., Zuccolo, L., Smith, G.D., Chen, L., Harris, R., Davis, M., Marsden, G., Johnston, C., Lane, J.A., Ebbing, M., Bønaa, K.H., Nygård, O., Ueland, P.M., Grau, M.V., Baron, J.A., Donovan, J.L., Neal, D.E., Hamdy, F.C., Smith, A.D., Martin, R.M., 2010. Circulating folate, vitamin B12, homocysteine, vitamin B12 transport proteins, and risk of prostate cancer: a case-control study, systematic review, and meta-analysis. Cancer Epidemiol Biomarkers Prev 19, 1632–1642.

Costa, D.S., Guahnon, M.P., Seganfredo, F.B., Pinto, L.P., Tovo, C.V., Fernandes, S.A., 2021. VITAMIN B12 AND HOMOCYSTEINE LEVELS IN PATIENTS WITH NAFLD: A SYSTEMATIC REVIEW AND METANALYSIS. Arq Gastroenterol 58, 234–239.

Danesh, J., Lewington, S., 1998. Plasma homocysteine and coronary heart disease: systematic review of published epidemiological studies. J Cardiovasc Risk 5, 229–232.

den Heijer, M., Rosendaal, F.R., Blom, H.J., Gerrits, W.B., Bos, G.M., 1998. Hyperhomocysteinemia and venous thrombosis: a meta-analysis. Thromb Haemost 80, 874–877.

Diao, J., Luo, L., Li, J., Zhang, S., Li, Y., Qin, J., 2020. Maternal homocysteine and folate levels and risk of recurrent spontaneous abortion: A meta-analysis of observational studies. Journal of Obstetrics and Gynaecology Research 46, 2461–2473.

Dong, X.J., Wang, B.B., Hou, F.F., Chen, K.P., Zhou, H.D., Guo, J.W., Sun, X., Liu, X.L., Chen, L.J., 2021. Homocysteine (HCY) levels in patients with atrial fibrillation (AF): A meta-analysis. International Journal of Clinical Practice 75.

Fan, R., Zhang, A., Zhong, F., 2017. Association between Homocysteine Levels and All-cause Mortality: A Dose-Response Meta-Analysis of Prospective Studies. Scientific reports 7, 4769.

Feng, Y., Shan, M.Q., Bo, L., Zhang, X.Y., Hu, J., 2015. Association of homocysteine with type 1 diabetes mellitus: A meta-analysis. International Journal of Clinical and Experimental Medicine 8, 12529–12538.

Ford, E.S., Smith, S.J., Stroup, D.F., Steinberg, K.K., Mueller, P.W., Thacker, S.B., 2002. Homocyst(e)ine and cardiovascular disease: a systematic review of the evidence with special emphasis on case-control studies and nested case-control studies. Int J Epidemiol 31, 59–70.

Fu, H.J., Zhao, L.B., Xue, J.J., Wu, Z.X., Huang, Y.P., Liu, W., Gao, Z., 2015. Elevated Serum Homocysteine (Hcy) Levels May Contribute to the Pathogenesis of Cerebral Infarction. Journal of Molecular Neuroscience 56, 553–561.

Gong, T., Wang, J., Yang, M., Shao, Y., Liu, J., Wu, Q., Xu, Q., Wang, H., He, X., Chen, Y., Xu, R., Wang, Y., 2016. Serum homocysteine level and gestational diabetes mellitus: A meta-analysis. Journal of Diabetes Investigation 7, 622–628.

Guo, B.Q., Li, H.B., Ding, S.B., 2020. Blood homocysteine levels in children with autism spectrum disorder: An updated systematic review and meta-analysis. Psychiatry Research 291.

Habib, S.S., Al-Khlaiwi, T., Almushawah, A., Alsomali, A., Habib, S.A., 2023. Homocysteine as a predictor and prognostic marker of atherosclerotic cardiovascular disease: a systematic review and meta-analysis. European Review for Medical and Pharmacological Sciences 27, 8598–8608.

He, J., Zhou, H., Xiong, J., Huang, Y., Huang, N., Jiang, J., 2024. Association between elevated homocysteine levels and obstructive sleep apnea hypopnea syndrome: a systematic review and updated meta-analysis. Frontiers in Endocrinology 15.

He, T., Jin, X., Koh, Y.S., Zhang, Q., Zhang, C., Liu, F., 2021. The association of homocysteine, folate, vitamin B12, and vitamin B6 with fracture incidence in older adults: A systematic review and meta-analysis. Annals of Translational Medicine 9.

Heinz, J., Kropf, S., Luley, C., Dierkes, J., 2009. Homocysteine as a risk factor for cardiovascular disease in patients treated by dialysis: a meta-analysis. Am J Kidney Dis 54, 478–489.

Ho, R.C., Cheung, M.W., Fu, E., Win, H.H., Zaw, M.H., Ng, A., Mak, A., 2011. Is high homocysteine level a risk factor for cognitive decline in elderly? A systematic review, meta-analysis, and meta-regression. Am J Geriatr Psychiatry 19, 607–617.

Hogeveen, M., Blom, H., Den Heijer, M., 2012. Maternal homocysteine in association with small for gestational age offspring: Systematic review and meta-analysis. Clinical Chemistry and Laboratory Medicine 50, A29.

Hu, N., Wang, X., 2023. The level of homocysteine in amyotrophic lateral sclerosis: a systematic review and meta-analysis. Neurological Sciences 44, 1185–1192.

Huang, P., Wang, F., Sah, B.K., Jiang, J., Ni, Z., Wang, J., Sun, X., 2015. Homocysteine and the risk of age-related macular degeneration: a systematic review and meta-analysis. Scientific reports 5, 10585.

Huang, T., Ren, J., Huang, J., Li, D., 2013. Association of homocysteine with type 2 diabetes: A meta-analysis implementing Mendelian randomization approach. BMC Genomics 14.

Huang, X., Yang, Y., Duan, Y., Kuang, Y.Q., Lin, D., 2017. Homocysteine in retinal artery occlusive disease: A meta-analysis of cohort studies. Scientific reports 7, 15708.

Jaiswal, V., Ang, S.P., Suresh, V., Joshi, A., Halder, A., Rajak, K., Nasir, Y.M., Gupta, S., Raj, N., Chia, J.E., Deb, N., Kainth, T., 2024. Association between baseline high-sensitive C-reactive protein, homocysteine levels, and post-stroke depression among stroke patients: a systematic review, meta-analysis, and meta-regression. Current Problems in Cardiology 49.

Jin, N., Huang, L., Hong, J., Zhao, X., Chen, Y., Hu, J., Cong, X., Xie, Y., Pu, J., 2021. Elevated homocysteine levels in patients with heart failure. Medicine (United States) 100.

Khandanpour, N., Loke, Y.K., Meyer, F.J., Jennings, B., Armon, M.P., 2009. Homocysteine and peripheral arterial disease: systematic review and meta-analysis. Eur J Vasc Endovasc Surg 38, 316–322.

Kim, J.E., Lee, H.J., Lee, J.S., Whang, K.U., Park, Y.L., Lee, S.Y., Kim, H.J., 2019. Association between homocysteine levels and psoriasis: A meta-analysis. Annals of Dermatology 31, 378–386.

Kumar, P., Nath, M., Misra, S., 2023. Elevated Homocysteine (Hcy) level as a risk factor for stroke and its subtype: A systematic review and meta-analysis. European Stroke Journal 8, 262.

La Regina, M., Orlandini, F., Prisco, D., Dentali, F., 2010. Homocysteine in vascular Behcet disease: a meta-analysis. Arterioscler Thromb Vasc Biol 30, 2067–2074.

Li, H., Shu, L., Dai, Q., Wu, T., 2022. Association between plasma total homocysteine (tHcy) and strokes: A meta-analysis. Pteridines 33, 58–68.

Li, H.H., Li, X.Q., Sai, L.T., Cui, Y., Xu, J.H., Zhou, C., Zheng, J., Li, X.F., Liu, H.X., Zhao, Y.J., 2021a. Association of homocysteine with ankylosing spondylitis: a systematic review and meta-analysis. Advances in Rheumatology 61.

Li, J., Feng, D., He, S., Wu, Q., Su, Z., Ye, H., 2021b. Meta-analysis: association of homocysteine with recurrent spontaneous abortion. Women & health 61, 713–720.

Li, J., Xu, F., Zeng, R., Gong, H., Lan, Y., 2016. Plasma homocysteine, serum folic acid, serum Vitamin B12, serum Vitamin B6, MTHFR, and risk of normal-tension glaucoma. Journal of Glaucoma 25, e94–e98.

Li, X., Yuan, J., Han, J., Hu, W., 2020. Serum levels of homocysteine, vitamin b12 and folate in patients with multiple sclerosis: An updated meta-analysis. International Journal of Medical Sciences 17, 751–761.

Liampas, I., Siokas, V., Mentis, A.A., Aloizou, A.M., Dastamani, M., Tsouris, Z., Aslanidou, P., Brotis, A., Dardiotis, E., 2020. Serum Homocysteine, Pyridoxine, Folate, and Vitamin B12 Levels in Migraine: Systematic Review and Meta-Analysis. Headache 60, 1508–1534.

Lin, X., Sun, T., Yu, R., Tang, Y., Li, H., 2016. Correlation between elevated serum homocysteine level and the development of diabetic peripheral neuropathy: A comparative study and meta-analysis. International Journal of Clinical and Experimental Medicine 9, 2857–2864.

Liu, Y., Gou, M., Guo, X., 2023. Features of Plasma Homocysteine, Vitamin B12, and Folate in Parkinson’s Disease: An Updated Meta-Analysis. Journal of Integrative Neuroscience 22.

Lu, X., Yuan, X., Chao, Y., Wu, X., Liu, A., 2024. The association of plasma homocysteine levels with short-term mortality in sepsis patients: A meta-analysis. Biomolecules & biomedicine.

Luo, H., Liu, B., Hu, J., Wang, X., Zhan, S., Kong, W., 2014. Hyperhomocysteinemia and methylenetetrahydrofolate reductase polymorphism in cervical artery dissection: A meta-analysis. Cerebrovascular Diseases 37, 313–322.

Mao, S., Xiang, W., Huang, S., Zhang, A., 2014. Association between homocysteine status and the risk of nephropathy in type 2 diabetes mellitus. Clinica Chimica Acta 431, 206–210.

Merashli, M., Bucci, T., Pastori, D., Pignatelli, P., Arcaro, A., Gentile, F., Marottoli, V., Ames, P.R.J., 2022. A Meta-Analysis of Plasma Homocysteine in Buerger's Disease. Thrombosis and Haemostasis 122, 1243–1246.

Moradi, F., Lotfi, K., Armin, M., Clark, C.C.T., Askari, G., Rouhani, M.H., 2021. The association between serum homocysteine and depression: A systematic review and meta-analysis of observational studies. European Journal of Clinical Investigation 51.

Muntjewerff, J.W., Kahn, R.S., Blom, H.J., den Heijer, M., 2006. Homocysteine, methylenetetrahydrofolate reductase and risk of schizophrenia: a meta-analysis. Mol Psychiatry 11, 143–149.

Nelen, W., Blom, H.J., Steegers, E.A.P., den Heijer, M., Eskes, T., 2000. Hyperhomocysteinemia and recurrent early pregnancy loss: a meta-analysis. Fertility and Sterility 74, 1196–1199.

Ni, G., Qin, J., Fang, Z., Chen, Y., Chen, Z., Zhou, J., Zhou, L., 2014. Increased homocysteine levels in valproate-treated patients with epilepsy: A meta-analysis. BMJ Open 4.

Niu, X., Chen, X., Xiao, Y., Dong, J., Zhang, R., Lu, M., Kong, W., 2014. The differences in homocysteine level between obstructive sleep apnea patients and controls: A meta-analysis. PLoS ONE 9.

Niu, X., Chen, Y., Zhong, Y., Xiao, X., 2023. The relationship between serum homocysteine levels and sudden sensorineural hearing loss: a meta-analysis. European Archives of Oto-Rhino-Laryngology 280, 2091–2097.

Oussalah, A., Guéant, J.L., Peyrin-Biroulet, L., 2011. Meta-analysis: hyperhomocysteinaemia in inflammatory bowel diseases. Aliment Pharmacol Ther 34, 1173–1184.

Peng, H.Y., Man, C.F., Xu, J., Fan, Y., 2015. Elevated homocysteine levels and risk of cardiovascular and all-cause mortality: a meta-analysis of prospective studies. Journal of Zhejiang University: Science B 16, 78–86.

Piao, X., Wu, G., Yang, P., Shen, J., De, A., Wu, J., Qu, Q., 2018. Association between Homocysteine and Cerebral Small Vessel Disease: A Meta-Analysis. Journal of Stroke and Cerebrovascular Diseases 27, 2423–2430.

Pinna, A., Zaccheddu, F., Boscia, F., Carru, C., Solinas, G., 2018. Homocysteine and risk of age-related macular degeneration: a systematic review and meta-analysis. Acta Ophthalmologica 96, e269–e276.

Rabelo, N.N., Telles, J.P.M., Pipek, L.Z., Farias Vidigal Nascimento, R., Gusmão, R.C., Teixeira, M.J., Figueiredo, E.G., 2022. Homocysteine is associated with higher risks of ischemic stroke: A systematic review and meta-analysis. PLoS ONE 17, e0276087.

Ray, J.G., 1998. Meta-analysis of hyperhomocysteinemia as a risk factor for venous thromboembolic disease. Arch Intern Med 158, 2101–2106.

Rong, H., Huang, L., Jin, N., Hong, J., Hu, J., Wang, S., Xie, Y., Pu, J., 2020. Elevated homocysteine levels associated with atrial fibrillation and recurrent atrial fibrillation. International Heart Journal 61, 705–712.

Sam, N.B., Zhang, Q., Li, B.Z., Li, X.M., Wang, D.G., Pan, H.F., Ye, D.Q., 2020. Serum/plasma homocysteine levels in patients with systemic lupus erythematosus: a systematic review and meta-analysis. Clinical Rheumatology 39, 1725–1736.

Sansone, A., Cignarelli, A., Romanelli, F., Corona, G., Gianfrilli, D., Isidori, A.M., Giorgino, F., 2019. PS-08-006 Serum homocysteine levels in men with and without erectile dysfunction: A systematic review and meta-analysis. Journal of Sexual Medicine 16, S27–S28.

Tian, X., Zheng, L., Leung, T.W., Leng, X., 2023. Associations of hematological and biochemical markers with intracranial atherosclerotic stenosis in stroke-free populations: A systematic review and meta-analysis of observational studies. Nutrition, Metabolism and Cardiovascular Diseases 33, 287–298.

Tsai, T.Y., Kuo, C.Y., Huang, Y.C., 2019a. Serum homocysteine, folate, and vitamin B12 levels in patients with vitiligo and their potential roles as disease activity biomarkers: A systematic review and meta-analysis. Journal of the American Academy of Dermatology 80, 646–654.e645.

Tsai, T.Y., Lee, T.H., Wang, H.H., Yang, T.H., Chang, I.J., Huang, Y.C., 2021. Serum Homocysteine, Folate, and Vitamin B12 Levels in Patients with Systemic Lupus Erythematosus: A Meta-Analysis and Meta-Regression. Journal of the American College of Nutrition 40, 443–453.

Tsai, T.Y., Yen, H., Huang, Y.C., 2019b. Serum homocysteine, folate and vitamin B12 levels in patients with psoriasis: a systematic review and meta-analysis. British Journal of Dermatology 180, 382–389.

Ulloque-Badaracco, J.R., Al-Kassab-Córdova, A., Alarcon-Braga, E.A., Hernandez-Bustamante, E.A., Huayta-Cortez, M.A., Cabrera-Guzmán, J.C., Robles-Valcarcel, P., Benites-Zapata, V.A., 2024a. Association of vitamin B12, folate, and homocysteine with COVID-19 severity and mortality: A systematic review and meta-analysis. SAGE Open Medicine 12.

Ulloque-Badaracco, J.R., Al-kassab-Córdova, A., Hernández-Bustamante, E.A., Alarcón-Braga, E.A., Cabrera-Guzmán, J.C., Horruitiner-Mendoza, A.A., Robles-Valcárcel, P., Benites-Zapata, V.A., Pérez-López, F.R., 2024b. Homocysteine, vitamin B12, and folate circulating levels in women with and without polycystic ovary syndrome: A systematic review and meta-analysis. Women's Health 20.

Ulloque-Badaracco, J.R., Alarcon-Braga, E.A., Hernandez-Bustamante, E.A., Von-Koeller-Jones, B.M., Huayta-Cortez, M., Saavedra-Custodio, E., Herrera-Añazco, P., Benites-Zapata, V.A., 2025. Vitamin B12, folate, and homocysteine levels in children and adolescents with obesity: a systematic review and meta-analysis. Front Public Health 13, 1481002.

Ulloque-Badaracco, J.R., Hernandez-Bustamante, E.A., Alarcon-Braga, E.A., Al-kassab-Córdova, A., Cabrera-Guzmán, J.C., Herrera-Añazco, P., Benites-Zapata, V.A., 2023. Vitamin B12, folate, and homocysteine in metabolic syndrome: a systematic review and meta-analysis. Frontiers in Endocrinology 14.

Unadkat, S.V., Padhi, B.K., Bhongir, A.V., Gandhi, A.P., Shamim, M.A., Dahiya, N., Satapathy, P., Rustagi, S., Khatib, M.N., Gaidhane, A., Zahiruddin, Q.S., Sah, R., Abu Serhan, H., 2024. Association between homocysteine and coronary artery disease-trend over time and across the regions: a systematic review and meta-analysis. Egyptian Heart Journal 76, 16.

Verkleij-Hagoort, A., Bliek, J., Sayed-Tabatabaei, F., Ursem, N., Steegers, E., Steegers-Theunissen, R., 2007. Hyperhomocysteinemia and MTHFR polymorphisms in association with orofacial clefts and congenital heart defects: a meta-analysis. Am J Med Genet A 143a, 952–960.

Visser, S., Hermes, W., Ket, J.C., Otten, R.H., van Pampus, M.G., Bloemenkamp, K.W., Franx, A., Mol, B.W., de Groot, C.J., 2014. Systematic review and metaanalysis on nonclassic cardiovascular biomarkers after hypertensive pregnancy disorders. Am J Obstet Gynecol 211, 373.e371–379.

Wald, D.S., Kasturiratne, A., Simmonds, M., 2011. Serum homocysteine and dementia: meta-analysis of eight cohort studies including 8669 participants. Alzheimers Dement 7, 412–417.

Wald, D.S., Law, M., Morris, J.K., 2004. The dose-response relation between serum homocysteine and cardiovascular disease: implications for treatment and screening. Eur J Cardiovasc Prev Rehabil 11, 250–253.

Wang, J., You, D., Wang, H., Yang, Y., Zhang, D., Lv, J., Luo, S., Liao, R., Ma, L., 2021a. Association between homocysteine and obesity: A meta-analysis. Journal of Evidence-Based Medicine 14, 208–217.

Wang, Q., Zhao, J., Chang, H., Liu, X., Zhu, R., 2021b. Homocysteine and Folic Acid: Risk Factors for Alzheimer's Disease—An Updated Meta-Analysis. Frontiers in Aging Neuroscience 13.

Wu, G., Xian, J., Yang, X., Li, J., Liu, J., Dong, W., Su, S., Li, J., Tu, Y., Peng, J., Xu, D., Zeng, Q., 2018. Association between homocysteine levels and calcific aortic valve disease: A systematic review and meta-analysis. Oncotarget 9, 8665–8674.

Xu, C., Wu, Y., Liu, G., Liu, X., Wang, F., Yu, J., 2014. Relationship between homocysteine level and diabetic retinopathy: a systematic review and meta-analysis. Diagnostic pathology 9, 167.

Xu, F., Zhang, L., Li, M., 2012a. Plasma homocysteine, serum folic acid, serum vitamin B12, serum vitamin B6, MTHFR and risk of pseudoexfoliation glaucoma: A meta-analysis. Graefe's Archive for Clinical and Experimental Ophthalmology 250, 1067–1074.

Xu, F., Zhao, X., Zeng, S.M., Li, L., Zhong, H.B., Li, M., 2012b. Homocysteine, B Vitamins, Methylenetetrahydrofolate Reductase Gene, and Risk of Primary Open-Angle Glaucoma <i>A Meta</i>-<i>analysis</i>. Ophthalmology 119, 2493–2499.

Xu, J., Zhao, X., Sun, S., Ni, P., Li, C., Ren, A., Wang, W., Zhu, L., 2018. Homocysteine and digestive tract cancer risk: A dose-response meta-analysis. Journal of Oncology 2018.

Yan, S., Liu, H., Yu, Y., Han, N., Du, W., 2022. Changes of Serum Homocysteine and Vitamin B12, but Not Folate Are Correlated With Obsessive-Compulsive Disorder: A Systematic Review and Meta-Analysis of Case-Control Studies. Frontiers in Psychiatry 13.

Yang, J., Hu, X., Zhang, Q., Cao, H., Wang, J., Liu, B., 2012. Homocysteine level and risk of fracture: A meta-analysis and systematic review. Bone 51, 376–382.

Yang, J., Li, H., Deng, H., Wang, Z., 2018. Association of one-carbon metabolism-related vitamins (Folate, B6, B12), homocysteine and methionine with the risk of lung cancer: Systematic review and meta-analysis. Frontiers in Oncology 8.

Yang, M., Li, W., Wan, Z., Du, Y., 2017. Elevated homocysteine levels in mothers with neural tube defects: a systematic review and meta-analysis. Journal of Maternal-Fetal and Neonatal Medicine 30, 2051–2057.

Yuan, F., Zheng, K., 2022. Homocysteine and the Mortality of Critically Ill Patients: A Meta-Analysis. Hormone and Metabolic Research 54, 593–603.

Yuan, S., Chen, J., Dan, L., Xie, Y., Sun, Y., Li, X., Larsson, S.C., 2022. Homocysteine, folate, and nonalcoholic fatty liver disease: a systematic review with meta-analysis and Mendelian randomization investigation. American Journal of Clinical Nutrition 116, 1595–1609.

Zhang, C., Hu, J., Wang, X., Gu, H., 2022. High level of homocysteine is associated with pre-eclampsia risk in pregnant woman: a meta-analysis. Gynecological Endocrinology 38, 705–712.

Zhang, D., Wen, X., Wu, W., Guo, Y., Cui, W., 2015. Elevated homocysteine level and folate deficiency associated with increased overall risk of carcinogenesis: Meta-analysis of 83 case-control studies involving 35,758 individuals. PLoS ONE 10.

Zhang, S.F., Li, L.Z., Zhang, W., Guo, J.R., Liu, F.F., Ma, K., Chen, S.H., Zhang, Y.Q., 2020. Association between Plasma Homocysteine Levels and Subclinical Hypothyroidism in Adult Subjects: A Meta-Analysis. Hormone and Metabolic Research 52, 625–638.

Zhang, Y.J., Zhang, L., Huang, X.L., Duan, Y., Yang, L.J., Wang, J., 2018. The association between homocysteine and systemic sclerosis: A review of the literature and meta-analysis. Modern Rheumatology 28, 681–689.

Zhang, Z., Xiao, S., Yang, C., Ye, R., Hu, X., Chen, X., 2019. Association of Elevated Plasma Homocysteine Level with Restenosis and Clinical Outcomes After Percutaneous Coronary Interventions: a Systemic Review and Meta-analysis. Cardiovascular Drugs and Therapy 33, 353–361.

Zhao, Y., Dong, X., Chen, B., Zhang, Y., Meng, S., Guo, F., Guo, X., Zhu, J., Wang, H., Cui, H., Li, S., 2022. Blood levels of circulating methionine components in Alzheimer’s disease and mild cognitive impairment: A systematic review and meta-analysis. Frontiers in Aging Neuroscience 14.

Zheng, Y., Deng, H.Y., Qiao, Z.Y., Gong, F.X., 2021. Homocysteine level and gestational diabetes mellitus: a systematic review and meta-analysis. Gynecological Endocrinology 37, 987–994.

Zhong, F., Zhuang, L., Wang, Y., Ma, Y., 2017. Homocysteine levels and risk of essential hypertension: A meta-analysis of published epidemiological studies. Clinical and Experimental Hypertension 39, 160–167.

Zhong, Y., Yan, F., Jie, W., Zhou, Y., Fang, F., 2019. Correlation between serum homocysteine level and ulcerative colitis: A meta-analysis. Pteridines 30, 114–120.

Zhou, F., Chen, S., 2019. Hyperhomocysteinemia and risk of incident cognitive outcomes: An updated dose-response meta-analysis of prospective cohort studies. Ageing Research Reviews 51, 55–66.

Zhou, Y., Chen, Y., Cao, X., Liu, C., Xie, Y., 2014. Association between plasma homocysteine status and hypothyroidism: A meta-analysis. International Journal of Clinical and Experimental Medicine 7, 4544–4553.

Zhou, Z., Liang, Y., Qu, H., Zhao, M., Guo, F., Zhao, C., Teng, W., 2018. Plasma homocysteine concentrations and risk of intracerebral hemorrhage: a systematic review and meta-analysis. Scientific reports 8, 2568.

Zinellu, A., Mangoni, A.A., 2023. Arginine, Transsulfuration, and Folic Acid Pathway Metabolomics in Chronic Obstructive Pulmonary Disease: A Systematic Review and Meta-Analysis. Cells 12.

**Supplementary Table 5. AMSTAR 2 quality assessment of meta-analyses included**

| **Authors, year** | **Title** | **1** | **2 b** | **3** | **4 b** | **5** | **6** | **7 b** | **8** | **9 b** | **10** | **11 b** | **12** | **13 b** | **14** | **15 b** | **16** | **Overall rating** |
| --- | --- | --- | --- | --- | --- | --- | --- | --- | --- | --- | --- | --- | --- | --- | --- | --- | --- | --- |
| The homocysteine studies collaboration 1997 | Homocysteine and risk of ischemic heart disease and stroke: a meta-analysis | yes | no | no | yes | no | no | no | yes | partial yes | no | yes | yes | no | yes | no | yes | **Critically low** |
| Bala 2021 | Hyperhomocysteinemia and low vitamin B12 are associated with the risk of early pregnancy loss: A clinical study and meta-analyses | no | no | yes | yes | no | no | no | partial yes | no | no | yes | no | no | no | no | yes | **Critically low** |
| Bautista 2002 | Total plasma homocysteine level and risk of cardiovascular disease: a meta-analysis of prospective cohort studies | no | no | yes | yes | no | no | yes | yes | yes | no | yes | yes | yes | yes | no | no | **Critically low** |
| Boushey 1995 | A meta-analysis of plasma homocysteine as a risk factor for arteriosclerotic vascular disease and the potential preventive role of folic acid | no | no | no | yes | no | no | no | no | no | no | yes | no | no | no | no | no | **Critically low** |
| Cahill 2003 | Meta-analysis of plasma homocysteine, serum folate, serum vitamin B<sub>12</sub> and thermolabile MTHFR genotype as risk factors for retinal vascular occlusive disease | yes | no | yes | yes | no | no | no | yes | no | no | yes | no | no | yes | no | no | **Critically low** |
| Cao 2014 | Homocysteine level and risk of abdominal aortic aneurysm: A meta-analysis | yer | no | yes | yes | yes | yes | partial yes | yes | no | no | yes | no | no | yes | no | yes | **Critically low** |
| Carpenè 2022 | Homocysteine in coronavirus disease (COVID-19): a systematic literature review | no | no | no | partial yes | no | no | no | yes | no | no | yes | no | no | no | no | yes | **Critically low** |
| Chen 2023 | Association of hyperhomocysteinemia and chronic kidney disease in the general population: a systematic review and meta-analysis | no | yes | yes | yes | yes | yes | yes | yes | yes | no | yes | yes | yes | yes | yes | yes | **Moderate** |
| Chen 2022 | Association between homocysteine levels in acute stroke and poststroke depression: A systematic review and meta-analysis | no | no | yes | yes | yes | yes | no | yes | no | no | yes | no | no | yes | no | yes | **Critically low** |
| Cheng 2024 | Association between serum homocysteine and erectile dysfunction: a systematic review and meta-analysis | yes | yes | yes | yes | yes | yes | yes | yes | yes | no | yes | yes | yes | no | no | yes | **Low** |
| Collin 2010 | Circulating folate, vitamin B12, homocysteine, vitamin B12 transport proteins, and risk of prostate cancer: a case-control study, systematic review, and meta-analysis | no | no | yes | yes | no | no | no | yes | no | no | yes | no | no | no | no | yes | **Critically low** |
| Costa 2021 | VITAMIN B12 AND HOMOCYSTEINE LEVELS IN PATIENTS WITH NAFLD: A SYSTEMATIC REVIEW AND METANALYSIS | no | yes | yes | yes | yes | yes | yes | yes | yes | no | yes | yes | no | yes | no | yes | **Critically low** |
| Danesh 1998 | Plasma homocysteine and coronary heart disease: systematic review of published epidemiological studies | no | no | yes | yes | no | no | no | yes | no | no | yes | no | no | no | no | no | **Critically low** |
| denHeijer 1998 | Hyperhomocysteinemia and venous thrombosis: a meta-analysis | no | no | yes | yes | no | no | no | yes | no | no | yes | no | no | no | no | no | **Critically low** |
| Diao 2020 | Maternal homocysteine and folate levels and risk of recurrent spontaneous abortion: A meta-analysis of observational studies | no | no | yes | yes | no | no | no | yes | yes | no | yes | no | no | yes | no | no | **Critically low** |
| Dong 2021 | Homocysteine (HCY) levels in patients with atrial fibrillation (AF): A meta-analysis | no | no | yes | yes | no | no | no | yes | no | no | yes | no | no | no | no | yes | **Critically low** |
| Fan 2017 | Association between Homocysteine Levels and All-cause Mortality: A Dose-Response Meta-Analysis of Prospective Studies | no | no | yes | yes | no | no | no | yes | no | no | yes | no | no | no | no | yes | **Critically low** |
| Feng 2015 | Association of homocysteine with type 1 diabetes mellitus: A meta-analysis | no | no | yes | yes | yes | yes | no | yes | no | no | yes | no | no | no | no | no | **Critically low** |
| Ford 2002 | Homocyst(e)ine and cardiovascular disease: a systematic review of the evidence with special emphasis on case-control studies and nested case-control studies | no | no | yes | yes | yes | no | no | yes | no | no | yes | no | no | no | no | no | **Critically low** |
| Fu 2015 | Elevated Serum Homocysteine (Hcy) Levels May Contribute to the Pathogenesis of Cerebral Infarction | no | no | yes | yes | no | no | no | yes | no | no | yes | no | no | no | no | yes | **Critically low** |
| Gong 2016 | Serum homocysteine level and gestational diabetes mellitus: A meta-analysis | no | no | yes | yes | yes | no | no | yes | no | no | yes | no | no | yes | no | yes | **Critically low** |
| Guo 2020 | Blood homocysteine levels in children with autism spectrum disorder: An updated systematic review and meta-analysis | no | no | yes | yes | yes | yes | yes | yes | yes | no | yes | yes | yes | yes | yes | yes | **Low** |
| Habib 2023 | Homocysteine as a predictor and prognostic marker of atherosclerotic cardiovascular disease: a systematic review and meta-analysis | yes | no | yes | yes | no | no | no | yes | no | no | yes | no | no | no | no | no | **Critically low** |
| He 2024 | Association between elevated homocysteine levels and obstructive sleep apnea hypopnea syndrome: a systematic review and updated meta-analysis | no | no | yes | yes | no | no | no | yes | no | no | yes | no | no | no | no | yes | **Critically low** |
| He 2021 | The association of homocysteine, folate, vitamin B12, and vitamin B6 with fracture incidence in older adults: A systematic review and meta-analysis | no | no | yes | yes | yes | yes | no | yes | yes | no | yes | yes | no | yes | no | yes | **Critically low** |
| Heinz 2009 | Homocysteine as a risk factor for cardiovascular disease in patients treated by dialysis: a meta-analysis | no | no | yes | yes | no | no | no | yes | no | no | yes | no | no | no | no | no | **Critically low** |
| Ho 2011 | Is high homocysteine level a risk factor for cognitive decline in elderly? A systematic review, meta-analysis, and meta-regression | no | no | yes | yes | no | no | yes | yes | yes | no | yes | yes | no | yes | no | yes | **Critically low** |
| Hogeveen 2012 | Maternal homocysteine in association with small for gestational age offspring: Systematic review and meta-analysis | no | no | yes | yes | no | no | no | yes | no | no | yes | no | no | no | no | no | **Critically low** |
| Hu 2023 | The level of homocysteine in amyotrophic lateral sclerosis: a systematic review and meta-analysis | no | no | yes | yes | yes | yes | no | yes | no | no | yes | no | no | no | no | yes | **Critically low** |
| Huang 2015 | Homocysteine and the risk of age-related macular degeneration: a systematic review and meta-analysis | no | no | yes | yes | no | no | no | yes | no | no | yes | no | no | no | no | yes | **Critically low** |
| Huang 2013 | Association of homocysteine with type 2 diabetes: A meta-analysis implementing Mendelian randomization approach | no | no | yes | yes | no | no | no | yes | yes | no | yes | no | no | yes | no | yes | **Critically low** |
| Huang 2017 | Homocysteine in retinal artery occlusive disease: A meta-analysis of cohort studies | no | no | yes | yes | no | no | yes | yes | yes | no | yes | yes | yes | yes | no | yes | **Critically low** |
| Jaiswal 2024 | Association between baseline high-sensitive C-reactive protein, homocysteine levels, and post-stroke depression among stroke patients: a systematic review, meta-analysis, and meta-regression | yes | no | yes | yes | no | no | no | yes | yes | no | yes | no | no | no | yes | yes | **Critically low** |
| Jin 2021 | Elevated homocysteine levels in patients with heart failure | no | no | yes | yes | yes | yes | no | yes | no | no | yes | no | no | no | no | yes | **Critically low** |
| Khandanpour 2009 | Homocysteine and peripheral arterial disease: systematic review and meta-analysis | no | no | yes | yes | no | no | no | yes | no | no | yes | no | no | no | no | yes | **Critically low** |
| Kim 2019 | Association between homocysteine levels and psoriasis: A meta-analysis | no | no | yes | yes | yes | yes | yes | yes | yes | no | yes | no | no | yes | no | yes | **Critically low** |
| Kumar 2023 | Elevated Homocysteine (Hcy) level as a risk factor for stroke and its subtype: A systematic review and meta-analysis | no | no | yes | yes | no | no | no | yes | yes | no | yes | no | no | yes | no | yes | **Critically low** |
| LaRegina 2010 | Homocysteine in vascular Behcet disease: a meta-analysis | no | no | yes | yes | yes | yes | no | yes | no | no | yes | no | no | no | no | yes | **Critically low** |
| Li 2022 | Association between plasma total homocysteine (tHcy) and strokes: A meta-analysis | no | no | yes | yes | yes | yes | no | yes | yes | no | yes | no | no | yes | no | yes | **Critically low** |
| Li 2021 | Association of homocysteine with ankylosing spondylitis: a systematic review and meta-analysis | no | no | yes | yes | yes | yes | no | yes | yes | no | yes | no | no | no | no | no | **Critically low** |
| Li 2021 | Meta-analysis: association of homocysteine with recurrent spontaneous abortion | no | no | yes | yes | yes | yes | no | yes | no | no | yes | no | no | no | no | yes | **Critically low** |
| Li 2016 | Plasma homocysteine, serum folic acid, serum Vitamin B12, serum Vitamin B6, MTHFR, and risk of normal-tension glaucoma | no | no | yes | yes | no | no | no | yes | no | no | yes | no | no | no | no | yes | **Critically low** |
| Li 2020 | Serum levels of homocysteine, vitamin b12 and folate in patients with multiple sclerosis: An updated meta-analysis | no | no | yes | yes | no | no | no | yes | yes | no | yes | no | no | yes | no | yes | **Critically low** |
| Liampas 2020 | Serum Homocysteine, Pyridoxine, Folate, and Vitamin B12 Levels in Migraine: Systematic Review and Meta-Analysis | no | no | yes | yes | no | no | no | yes | no | no | yes | no | no | no | no | yes | **Critically low** |
| Lin 2016 | Correlation between elevated serum homocysteine level and the development of diabetic peripheral neuropathy: A comparative study and meta-analysis | no | no | yes | yes | no | no | no | yes | no | no | yes | no | no | no | no | no | **Critically low** |
| Liu 2023 | Features of Plasma Homocysteine, Vitamin B12, and Folate in Parkinson’s Disease: An Updated Meta-Analysis | no | no | yes | yes | no | no | no | yes | yes | no | yes | yes | no | yes | no | yes | **Critically low** |
| Lu 2024 | The association of plasma homocysteine levels with short-term mortality in sepsis patients: A meta-analysis | no | no | yes | yes | no | no | no | yes | no | no | yes | no | no | no | no | yes | **Critically low** |
| Luo 2014 | Hyperhomocysteinemia and methylenetetrahydrofolate reductase polymorphism in cervical artery dissection: A meta-analysis | no | no | yes | yes | no | no | no | yes | no | no | yes | no | no | no | no | yes | **Critically low** |
| Mao 2014 | Association between homocysteine status and the risk of nephropathy in type 2 diabetes mellitus | no | no | yes | yes | no | no | no | yes | no | no | yes | no | no | no | no | yes | **Critically low** |
| Merashli 2022 | A Meta-Analysis of Plasma Homocysteine in Buerger's Disease | no | no | no | yes | no | no | no | yes | no | no | yes | no | no | no | no | yes | **Critically low** |
| Moradi 2021 | The association between serum homocysteine and depression: A systematic review and meta-analysis of observational studies | no | no | yes | yes | no | no | no | yes | no | no | yes | no | no | no | no | no | **Critically low** |
| Muntjewerff 2006 | Homocysteine, methylenetetrahydrofolate reductase and risk of schizophrenia: a meta-analysis | no | no | yes | yes | no | no | no | yes | no | no | yes | no | no | no | no | no | **Critically low** |
| Nelen 2000 | Hyperhomocysteinemia and recurrent early pregnancy loss: a meta-analysis | no | no | no | yes | no | no | no | yes | no | no | yes | no | no | no | no | no | **Critically low** |
| Ni 2014 | Increased homocysteine levels in valproate-treated patients with epilepsy: A meta-analysis | no | no | yes | yes | no | no | no | yes | no | no | yes | no | no | no | no | yes | **Critically low** |
| Niu 2014 | The differences in homocysteine level between obstructive sleep apnea patients and controls: A meta-analysis | no | no | yes | yes | no | no | no | yes | yes | no | yes | no | no | no | no | no | **Critically low** |
| Niu 2023 | The relationship between serum homocysteine levels and sudden sensorineural hearing loss: a meta-analysis | no | no | yes | yes | no | no | no | yes | yes | no | yes | no | no | no | no | yes | **Critically low** |
| Oussalah 2011 | Meta-analysis: hyperhomocysteinaemia in inflammatory bowel diseases | no | no | no | yes | no | no | no | yes | no | no | yes | no | no | no | no | no | **Critically low** |
| Peng 2015 | Elevated homocysteine levels and risk of cardiovascular and all-cause mortality: a meta-analysis of prospective studies | no | no | yes | yes | yes | yes | no | yes | no | no | yes | no | no | no | no | no | **Critically low** |
| Piao 2018 | Association between Homocysteine and Cerebral Small Vessel Disease: A Meta-Analysis | no | no | yes | yes | no | no | no | yes | yes | no | yes | no | no | no | no | no | **Critically low** |
| Pinna 2018 | Homocysteine and risk of age-related macular degeneration: a systematic review and meta-analysis | no | no | yes | yes | yes | no | no | yes | no | no | yes | no | no | no | no | no | **Critically low** |
| Rabelo 2022 | Homocysteine is associated with higher risks of ischemic stroke: A systematic review and meta-analysis | no | no | yes | yes | yes | no | no | yes | no | no | yes | no | no | no | no | no | **Critically low** |
| Ray 1998 | Meta-analysis of hyperhomocysteinemia as a risk factor for venous thromboembolic disease | no | no | yes | yes | no | no | no | yes | no | no | yes | no | no | no | no | no | **Critically low** |
| Rong 2020 | Elevated homocysteine levels associated with atrial fibrillation and recurrent atrial fibrillation | no | no | yes | yes | yes | no | no | yes | no | no | yes | no | no | no | no | yes | **Critically low** |
| Sam 2020 | Serum/plasma homocysteine levels in patients with systemic lupus erythematosus: a systematic review and meta-analysis | no | no | yes | yes | yes | no | no | yes | no | no | yes | no | no | no | no | yes | **Critically low** |
| Sansone 2019 | PS-08-006 Serum homocysteine levels in men with and without erectile dysfunction: A systematic review and meta-analysis | no | no | yes | yes | yes | yes | no | yes | no | no | yes | no | no | no | no | yes | **Critically low** |
| Tian 2023 | Associations of hematological and biochemical markers with intracranial atherosclerotic stenosis in stroke-free populations: A systematic review and meta-analysis of observational studies | no | no | yes | yes | yes | yes | no | yes | yes | no | yes | yes | no | no | no | yes | **Critically low** |
| Tsai 2019 | Serum homocysteine, folate, and vitamin B12 levels in patients with vitiligo and their potential roles as disease activity biomarkers: A systematic review and meta-analysis | no | no | yes | yes | yes | yes | no | yes | no | no | yes | no | no | no | no | yes | **Critically low** |
| Tsai 2021 | Serum Homocysteine, Folate, and Vitamin B12 Levels in Patients with Systemic Lupus Erythematosus: A Meta-Analysis and Meta-Regression | no | no | yes | yes | yes | yes | no | yes | no | no | yes | no | no | no | no | yes | **Critically low** |
| Tsai 2019 | Serum homocysteine, folate and vitamin B12 levels in patients with psoriasis: a systematic review and meta-analysis | no | no | yes | yes | yes | yes | no | yes | no | no | yes | no | no | no | no | no | **Critically low** |
| Ulloque-Badaracco 2024 | Association of vitamin B12, folate, and homocysteine with COVID-19 severity and mortality: A systematic review and meta-analysis | no | no | yes | yes | no | no | no | yes | no | no | yes | no | no | no | no | yes | **Critically low** |
| Ulloque-Badaracco 2024 | Homocysteine, vitamin B12, and folate circulating levels in women with and without polycystic ovary syndrome: A systematic review and meta-analysis | no | no | yes | yes | no | no | no | yes | yes | no | yes | no | no | yes | no | yes | **Critically low** |
| Ulloque-Badaracco 2025 | Vitamin B12, folate, and homocysteine levels in children and adolescents with obesity: a systematic review and meta-analysis | no | no | yes | yes | yes | yes | no | yes | yes | no | yes | yes | yes | no | no | yes | **Critically low** |
| Ulloque-Badaracco 2023 | Vitamin B12, folate, and homocysteine in metabolic syndrome: a systematic review and meta-analysis | no | no | yes | yes | yes | yes | no | yes | yes | no | yes | no | no | no | yes | yes | **Critically low** |
| Unadkat 2024 | Association between homocysteine and coronary artery disease-trend over time and across the regions: a systematic review and meta-analysis | no | no | yes | yes | yes | yes | no | yes | yes | no | yes | no | no | no | yes | yes | **Critically low** |
| Verkleij-Hagoort 2007 | Hyperhomocysteinemia and MTHFR polymorphisms in association with orofacial clefts and congenital heart defects: a meta-analysis | no | no | yes | yes | no | no | no | yes | no | no | yes | no | no | no | no | no | **Critically low** |
| Visser 2014 | Systematic review and metaanalysis on nonclassic cardiovascular biomarkers after hypertensive pregnancy disorders | no | no | yes | yes | yes | yes | no | yes | no | no | yes | no | no | no | no | no | **Critically low** |
| Wald 2011 | Serum homocysteine and dementia: meta-analysis of eight cohort studies including 8669 participants | no | no | yes | yes | no | no | no | yes | no | no | yes | no | no | no | no | no | **Critically low** |
| Wald 2004 | The dose-response relation between serum homocysteine and cardiovascular disease: implications for treatment and screening | no | no | yes | yes | no | no | yes | yes | no | no | yes | no | no | no | no | no | **Critically low** |
| Wang 2021 | Association between homocysteine and obesity: A meta-analysis | no | no | yes | yes | yes | yes | no | yes | yes | no | yes | yes | yes | no | yes | no | **Critically low** |
| Wang 2021 | Homocysteine and Folic Acid: Risk Factors for Alzheimer's Disease—An Updated Meta-Analysis | no | no | yes | yes | yes | no | no | yes | no | no | yes | no | no | no | no | no | **Critically low** |
| Wu 2018 | Association between homocysteine levels and calcific aortic valve disease: A systematic review and meta-analysis | no | no | yes | yes | no | no | no | yes | yes | no | yes | yes | yes | no | yes | yes | **Critically low** |
| Xu 2014 | Relationship between homocysteine level and diabetic retinopathy: a systematic review and meta-analysis | no | no | yes | yes | yes | yes | no | yes | no | no | yes | no | no | no | no | no | **Critically low** |
| Xu 2012 | Plasma homocysteine, serum folic acid, serum vitamin B12, serum vitamin B6, MTHFR and risk of pseudoexfoliation glaucoma: A meta-analysis | no | no | yes | yes | yes | yes | no | yes | no | no | yes | no | no | no | no | no | **Critically low** |
| Xu 2012 | Homocysteine, B Vitamins, Methylenetetrahydrofolate Reductase Gene, and Risk of Primary Open-Angle Glaucoma <i>A Meta</i>-<i>analysis</i> | no | no | yes | yes | yes | yes | no | yes | no | no | yes | no | no | no | no | yes | **Critically low** |
| Xu 2018 | Homocysteine and digestive tract cancer risk: A dose-response meta-analysis | no | no | yes | yes | yes | yes | no | yes | no | no | yes | no | no | no | no | yes | **Critically low** |
| Yan 2022 | Changes of Serum Homocysteine and Vitamin B12, but Not Folate Are Correlated With Obsessive-Compulsive Disorder: A Systematic Review and Meta-Analysis of Case-Control Studies | no | no | yes | yes | yes | yes | no | yes | yes | no | yes | no | no | no | no | yes | **Critically low** |
| Yang 2012 | Homocysteine level and risk of fracture: A meta-analysis and systematic review | no | no | yes | yes | yes | yes | no | yes | no | no | yes | no | no | no | no | yes | **Critically low** |
| Yang 2018 | Association of one-carbon metabolism-related vitamins (Folate, B6, B12), homocysteine and methionine with the risk of lung cancer: Systematic review and meta-analysis | no | no | yes | yes | yes | yes | no | yes | no | no | yes | no | no | no | yes | no | **Critically low** |
| Yang 2017 | Elevated homocysteine levels in mothers with neural tube defects: a systematic review and meta-analysis | no | no | yes | yes | yes | yes | no | yes | no | no | yes | no | no | no | no | yes | **Critically low** |
| Yuan 2022 | Homocysteine and the Mortality of Critically Ill Patients: A Meta-Analysis | no | no | yes | yes | yes | yes | no | yes | yes | no | yes | no | no | no | yes | yes | **Critically low** |
| Yuan 2022 | Homocysteine, folate, and nonalcoholic fatty liver disease: a systematic review with meta-analysis and Mendelian randomization investigation | no | no | yes | yes | yes | yes | no | yes | no | no | yes | no | no | no | no | no | **Critically low** |
| Zhang 2022 | High level of homocysteine is associated with pre-eclampsia risk in pregnant woman: a meta-analysis | no | no | yes | yes | yes | no | no | yes | yes | no | yes | yes | no | no | yes | yes | **Critically low** |
| Zhang 2015 | Elevated homocysteine level and folate deficiency associated with increased overall risk of carcinogenesis: Meta-analysis of 83 case-control studies involving 35,758 individuals | no | no | yes | yes | no | no | no | yes | no | no | yes | no | no | no | no | yes | **Critically low** |
| Zhang 2020 | Association between Plasma Homocysteine Levels and Subclinical Hypothyroidism in Adult Subjects: A Meta-Analysis | no | no | yes | yes | yes | yes | no | yes | no | no | yes | no | no | no | no | yes | **Critically low** |
| Zhang 2018 | The association between homocysteine and systemic sclerosis: A review of the literature and meta-analysis | no | no | no | yes | no | no | no | yes | no | no | yes | no | no | no | no | yes | **Critically low** |
| Zhang 2019 | Association of Elevated Plasma Homocysteine Level with Restenosis and Clinical Outcomes After Percutaneous Coronary Interventions: a Systemic Review and Meta-analysis | no | no | yes | yes | yes | no | no | yes | no | no | yes | no | no | no | no | yes | **Critically low** |
| Zhao 2022 | Blood levels of circulating methionine components in Alzheimer’s disease and mild cognitive impairment: A systematic review and meta-analysis | no | no | yes | yes | yes | yes | no | yes | yes | no | yes | no | no | no | yes | yes | **Critically low** |
| Zheng 2021 | Homocysteine level and gestational diabetes mellitus: a systematic review and meta-analysis | no | no | yes | yes | yes | no | no | yes | no | no | yes | no | no | no | no | yes | **Critically low** |
| Zhong 2017 | Homocysteine levels and risk of essential hypertension: A meta-analysis of published epidemiological studies | no | no | yes | yes | yes | yes | no | yes | yes | no | yes | no | no | no | yes | yes | **Critically low** |
| Zhong 2019 | Correlation between serum homocysteine level and ulcerative colitis: A meta-analysis | no | no | yes | yes | yes | no | no | yes | no | no | yes | no | no | no | yes | yes | **Critically low** |
| Zhou 2019 | Hyperhomocysteinemia and risk of incident cognitive outcomes: An updated dose-response meta-analysis of prospective cohort studies | no | no | yes | yes | no | no | no | yes | no | no | yes | no | no | no | no | yes | **Critically low** |
| Zhou 2014 | Association between plasma homocysteine status and hypothyroidism: A meta-analysis | no | no | yes | yes | no | no | no | yes | no | no | yes | no | no | no | no | no | **Critically low** |
| Zhou 2018 | Plasma homocysteine concentrations and risk of intracerebral hemorrhage: a systematic review and meta-analysis | no | no | yes | yes | yes | no | no | yes | no | no | yes | no | no | no | no | yes | **Critically low** |
| Zhou 2021 | Serum Uric Acid and the Risk of Dementia: A Systematic Review and Meta-Analysis | no | no | yes | yes | yes | yes | no | yes | yes | no | yes | no | no | no | yes | yes | **Critically low** |
| Zinellu 2023 | Arginine, Transsulfuration, and Folic Acid Pathway Metabolomics in Chronic Obstructive Pulmonary Disease: A Systematic Review and Meta-Analysis | no | no | yes | yes | no | no | no | yes | no | no | yes | no | no | no | no | yes | **Critically low** |

Notes:

1.	Did the research questions and inclusion criteria for the review include the components of PICO (Population, Intervention, Comparator group, Outcome)? YES/NO. For yes, must have all four.

2.	Did the report of the review contain an explicit statement that the review methods were established prior to the conduct of the review and did the report justify any significant deviations from the protocol? YES, PARTIAL YES, NO. For Partial YES: the authors state that they had a written protocol or guide that included ALL the following (review question(s), a search strategy, inclusion/exclusion criteria, a risk of bias assessment). For YES: as for partial yes, plus the protocol should be registered and should also have specified: a meta-analysis/synthesis plan, if appropriate, and a plan for investigating causes of heterogeneity, justification for any deviations from the protocol.

3.	Did the review authors explain their selection of the study designs for inclusion in the review? YES/NO. For YES, the review should satisfy one of the following: explanation for including only RCTs, or explanation for including only NRSI, or explanation for including both RCTs and NRSI.

4.	Did the review authors use a comprehensive literature search strategy? YES, PARTIAL YES, NO. for PARTIAL YES must have all of the following: searched at least 2 databases (relevant to research question), provided key word and/or search strategy, justified publication restrictions (eg. Language). For YES should also have all of the following: searched the reference lists/biographies of included studies, searched trial/study registries, included/consulted content experts in the field, searched for grey literature where relevant, conducted search within 24 months of completion of the review.

5.	Did the review authors perform study selection in duplicate? YES/NO. for YES, either ONE of the following: at least two reviewers independently agreed on selection of eligible studies and achieved consensus on which studies to include OR two reviewers selected a sample of eligible studies and achieved good agreement (at least 80 per cent) with the remainder selected by one reviewer.

6.	Did the review authors perform data extraction in duplicate? YES/NO. For YES, either one of the following: at least two reviewers achieved consensus on which data to extract from included studies OR two reviewers extracted data from a sample of eligible studies and achieved good agreement (at least 80 per cent) with the remainder extracted by one reviewer.

7.	Did the review authors provide a list of excluded studies to justify the exclusions? YES, PARTIAL YES, NO. FOR partial yes must provide a list of all potentially relevant studies that were read in full text form but excluded from the review. For YES must also have justified the exclusion from the review of each potentially relevant study.

8.	Did the review authors describe the included studies in adequate detail? YES, PARTIAL YES, NO. For PARTIAL YES, must describe all of the following: populations, interventions, comparators, outcomes, research designs. For YES should also have all of the following: described populations in detail, described intervention and comparator in detail (including doses where relevant), described study setting, timeframe or follow-up.

9.	Did the review authors use a satisfactory technique for assessing the risk of bias (RoB) in individual studies that were included in the review? For RCTs: YES, PARTIAL YES, NO, INCLUDES ONLY NRSI. For PARTIAL YES must have assessed RoB from unconcealed allocation and lack of blinding of patients and assessors when assessing outcomes (unnecessary for objective outcomes such as all cause mortality); for YES must also have assessed RoB from allocation sequence that was not truly random and selection of the reported result from among multiple measurements or analyses of a specified outcome. For NRSI (Non Randomized Studies of Intervention): YES, PARTIAL YES, NO, INCLUDES ONLY RCTs. For PARTIAL YES must have assessed RoB from confounding and from selection bias. For YES, must also have assessed methods used to ascertain exposures and outcomes, and selection of the reported results from among multiple measurements or analyses of a specified outcome.

10.	Did the review authors report on the sources of funding for the studies included in the review? YES/NO. For YES: must have reported on the sources of funding for individual studies included in the review. Note: reporting that the reviewers looked for this information but it was not reported by study authors also qualifies

11.	If meta-analysis was performed, did the review authors use appropriate methods for statistical combination of results? For RCTs: YES, NO, NO META-ANALYSIS. For YES: the authors justified combining the data in a meta-analysis and they used an appropriate weighted technique to combine study results and adjusted for heterogeneity if present and investigated the causes of heterogeneity. For NRSI: YES, NO, NO META-ANALYSIS CONDUCTED. For YES: the authors justified combining the data in a meta-analysis and they used an appropriate weighted technique to combine study results, adjusting for heterogeneity if present, and they statistically combined effects estimates from NRSI that were adjusted for confounding, rather than combining raw data, or justified combining raw data when adjusted effect estimates were not available, and they reported separate summary estimates for RCTs and NRSI separately when both were included in the review.

12.	If meta-analysis was performed, did the review authors assess the potential impact of RoB in individual studies on the results of the meta-analysis or other evidence synthesis? YES, NO, NO META-ANALYSIS INCLUDED. For YES: included only low risk of bias RCTs or, if the pooled estimate was based on RCTs and/or NRSI at variable RoB, the authors performed analysis ton investigate possible impact of RoB on summary estimates of effect.

13.	Did the review authors account for RoB in individual studies when interpreting/discussing the results of the review? YES/NO. for YES: included only low risk of bias RCTs or, if RCTs with moderate or high RoB, or NRSI were included, the review provided a discussion of the key impact of RoB on the results

14.	Did the review authors provide a satisfactory explanation for, and discussion of, any heterogeneity observed in the results of the review? YES/NO. For Yes: there was no significant heterogeneity in the results OR if heterogeneity was present the authors performed an investigation of sources of any heterogeneity in the results and discussed the impact of this on the results of the review

15.	If they performed quantitative synthesis did the review authors carry out an adequate investigation of publication bias (small study bias) and discuss its likely impact on the results of the review? YES, NO, NO META-ANALYSIS CONDUCTED. For YES: performed graphical statistical tests for publication bias and discussed the likelihood and magnitude of impact of publication bias

16.	Did the review authors report any potential sources of conflict of interest, including any funding they received for conducting the review? YES/NO. For Yes: the authors reported no competing interests OR the authors described their funding sources and how they managed potential conflicts of interest.

d Rating overall confidence in the results of the review:

HIGH: no on one non-critical weakness: the systematic review provides an accurate and comprehensive summary of the results of the available studies that address the question of interest

MODERATE: more than one non critical weakness (multiple non-critical weaknesses may diminish confidence in the review and it may be appropriate to move the overall appraisal down from moderate to low confidence): the systematic review has more than one weakness but no critical flaws. It may provide an accurate summary of the results of the available studies that were included in the review

LOW: one critical flaw with or without non-critical weaknesses: the review has a critical flaw and may not provide an accurate and comprehensive summary of the available studies that address the question of interest

CRITICALLY LOW: more than one critical flaw with or without non-critical weaknesses: the review has more than one critical flaw and should not be relied on to provide an accurate and comprehensive summary of the available studies.
